# Biofilm-mediated antibiotic cross-protection: *Acinetobacter baumannii*-driven enhancement of *Elizabethkingia anopheles*

**DOI:** 10.1128/spectrum.03349-25

**Published:** 2026-06-16

**Authors:** Dejin Pan, Ren Liu, Jiehui Qiu, Ziyi Wu, Nan Su, Jianglong Shi, Na Cheng

**Affiliations:** 1 Department of Infectious Disease, The First Affiliated Hospital, Jiangxi Medical College, Nanchang University 74653, Nanchang, Jiangxi, China; 2 Jiangxi Medical Center for Critical Public Health Events, The First Affiliated Hospital, Jiangxi Medical College, Nanchang University 74653, Nanchang, Jiangxi, China; 3 Departments of Hospital Infection Control, the First Affiliated Hospital, Jiangxi Medical College, Nanchang University 74653, Nanchang, Jiangxi, China; Rowan University Cooper Medical School, Camden, New Jersey, USA; Medical City Campus/Medical Microbiology Teaching Laboratories, Baghdad, Iraq; Osmania University, Hyderabad, India

**Keywords:** coinfection, biofilm formation, cross-protection, *Acinetobacter baumannii*, *Elizabethkingia anophelis*

## Abstract

*Acinetobacter baumannii* is a clinically prevalent multidrug-resistant bacterium, while *Elizabethkingia*, an emerging pathogen, has shown increasing infection and mortality rates in recent years. Although co-infections involving both pathogens have been reported, their interactions remain poorly understood. In this study, clinical data from 15 co-infected patients revealed that such infections were more frequent in elderly individuals with underlying diseases requiring intensive care and prolonged hospitalization. The two isolates exhibited similar antibiotic resistance profiles. *In vitro* co-culture experiments showed that *Elizabethkingia anophelis* alone was sensitive to doxycycline, but after co-culturing with *A. baumannii*, it survived at concentrations over 16 times its minimum inhibitory concentration (MIC), suggesting that *A. baumannii* may mediate antibiotic cross-protection. The maximum biomass (OD570) of the co-culture exceeded that of the monoculture under multiple antibiotic conditions. Transcriptomic analysis revealed no significant changes in drug resistance genes in either strain, but *A. baumannii* showed strong upregulation of the biofilm-associated gene *pgaB*. GO and KEGG analyses indicated enrichment in biofilm formation, adhesion, and exopolysaccharide synthesis. In contrast, *E. anophelis* showed reduced expression of its biofilm-related genes. Crystal violet staining, confocal microscopy, and SEM confirmed that co-culturing enhanced and compacted biofilm structure. Moreover, the *Galleria mellonella* model demonstrated higher virulence in co-infections. These findings suggest that biofilm enhancement mediated by *A. baumannii* promotes antibiotic tolerance in *Elizabethkingia*. This study provides new insights into polymicrobial co-infections and supports the development of therapeutic strategies targeting interspecies interactions.

**IMPORTANCE:**

Hospital infections often involve more than one kind of bacterium, making them harder to treat. In this study, we focused on *Acinetobacter baumannii*, a well-known drug-resistant hospital pathogen, and *Elizabethkingia anophelis*, an emerging bacterium linked to severe infections. By analyzing patients and conducting laboratory experiments, we found that when these two bacteria grow together, *E. anophelis* becomes more tolerant to antibiotics and the mixed community forms stronger biofilms—protective layers that help bacteria survive. This cooperation also increased infection severity in an insect model. Our findings suggest that *A. baumannii* can enhance the persistence and drug resistance of *E. anophelis* through biofilm-related interactions. Understanding such bacterial partnerships may help develop better ways to control hospital infections and guide new antimicrobial strategies.

## INTRODUCTION

*Acinetobacter baumannii* is one of the most successful pathogens causing nosocomial infections in modern healthcare systems. It often coexists with other drug-resistant bacteria, such as *Klebsiella pneumoniae* and *Pseudomonas aeruginosa*, significantly increasing patient mortality rates (1–3). *A. baumannii* has been classified as one of the ESKAPE pathogens. *Elizabethkingia*, as an emerging pathogen, is receiving increasing attention (4). It is widely distributed in the environment and can cause various diseases such as bloodstream infections, meningitis, endocarditis, pneumonia, endophthalmitis, and urinary tract infections, with a mortality rate of 24%–60% when infected alone (5, 6). This opportunistic pathogen is capable of forming biofilms, surviving for extended periods in moist environments, and colonizing healthcare settings readily (7–9). Hospital reports indicate that the infection rate of *Elizabethkingia* among hospitalized patients increased from 0.02‰ in 2009 to 0.88‰ in 2017, with its incidence continuing to rise in recent years, often leading to severe clinical outcomes (10–13). As a significant pathogen in hospital-acquired infections, clinical observations have revealed co-infections involving *A. baumannii* and *Elizabethkingia*, presenting complex clinical changes (14). However, how the antibiotic resistance mechanisms interact with and influence virulence changes following co-infection remains poorly understood.

Polymicrobial infections caused by two or more pathogens are common, yet current scientific research predominantly concentrates on the pathogenic mechanisms of individual pathogens, resulting in a limited understanding of the interactions and mechanisms that underlie co-infections involving multiple pathogens. In the microenvironment of most hosts, polymicrobial infections frequently exhibit synergistic effects (15–17). The expression of microbial virulence factors, exchange of signaling molecules, adaptive regulation of metabolism, and transmission of antibiotic resistance may all be influenced by the social regulation of coexisting microorganisms (18, 19), thereby impacting the bacteria’s ability to adapt to changing environments (20, 21). Evidence indicates that, in comparison to single infections, co-infections can lead to heightened antibiotic resistance and increased mortality rates in the host (22–24). For example, in patients with cystic fibrosis (CF), co-infection with *Pseudomonas aeruginosa* and *Staphylococcus aureus* results in more severe pulmonary inflammation than infections caused by a single pathogen (25). Similarly, in patients positive for carbapenem-resistant Enterobacteriaceae (CRE), co-infection with *A. baumannii* and/or *P. aeruginosa* exacerbates both antibiotic resistance and mortality rates (26). Furthermore, co-infection with influenza A virus and *Streptococcus pneumoniae* is often associated with elevated mortality rates (27).

The mechanisms underlying polymicrobial infections in hosts remain poorly understood, presenting significant challenges for clinical treatment. This study investigates *A. baumannii* and *Elizabethkingia*, which were isolated from a patient with a pulmonary infection, focusing on their interactions concerning virulence and drug resistance. We retrospectively analyzed the clinical data of 15 patients co-infected with *A. baumannii* and *Elizabethkingia* at the First Affiliated Hospital of Nanchang University from March 2024 to March 2025. Based on the clinical and microbiological characteristics observed in the cohort, a representative strain pair was selected for *in vitro* co-culture experiments to investigate their interactions in terms of virulence, antimicrobial resistance, and other relevant aspects, with the aim of elucidating the potential impact of their coexistence within the host environment. Additionally, we performed DNA sequencing and genomic analysis to characterize the genomes, virulence factors, and antibiotic resistance profiles of these bacterial strains and conducted transcriptomic analysis on the co-cultures, comparing their gene expression profiles with those observed in their respective monocultures.

## RESULTS

### Clinical data

We collected data from the medical record system of the First Affiliated Hospital of Nanchang University, involving 15 cases of patients co-infected with *A. baumannii* and *Elizabethkingia*. All co-infection cases were identified from the same patient during a single hospitalization, with the time interval between the isolation of the two strains not exceeding 3 days. The patients exhibited persistent or worsening clinical symptoms, necessitating antimicrobial treatment based on culture results. The collected information encompassed sex, age, comorbidities, primary diagnosis, specimen source, ICU admission, performance of invasive procedures such as tracheal intubation, length of hospital stay, outcome (Table 1), and antimicrobial susceptibility profiles of the two isolated strains (Table 2).

**TABLE 1:** Clinical characteristics of 15 patients with *A. baumannii* and *Elizabethkingia* co-infection

Variable	Value
Male sex (%)	11/15 (73%)
Age, median (IQR)	70 (56–79) years
Comorbidity, ≥1 (%)	13/15 (86%)
ICU admission (%)	15/15 (100%)
Invasive procedures (%)	15/15 (100%)
Hospital stay, median (IQR)	34 (25–56) days

**TABLE 2:** Antibiotic susceptibility patterns of *A. baumannii* and *Elizabethkingia* isolates (*n* = 15)^*a*^

Antibiotic	*A. baumannii*, % resistant	*Elizabethkingia*, % resistant
Ceftazidime	R 100% (15/15)	R 100% (15/15)
Cefepime	R 80%(12/15)	R 100% (15/15)
Piperacillin–tazobactam	R 100% (15/15)	R 93% (14/15)
Cefoperazone–sulbactam	R 73% (11/15)	NA
Meropenem	R 100% (15/15)	R 100% (15/15)
Imipenem	R 100% (15/15)	R 100% (15/15)
Doxycycline	R 66% (10/15)	S 93% (14/15)
Tigecycline	S 100% (15/15)	NA
Colistin	S 93% (14/15)	NA
Minocycline	S 60% (9/15)	S 100% (15/15)
Ciprofloxacin	R 100% (15/15)	R 60% (9/15)
Amikacin	R 93% (14/15)	R 100% (15/15)
Tobramycin	R 86% (13/15)	R 100% (15/15)
Aztreonam	NA	R 100% (15/15)

^*a*^ R, Resistant; S, Sensitive; I, Intermediate; NA, not available/applicable. Antimicrobial susceptibility was interpreted according to CLSI guidelines.

Among the 15 patients, the majority were elderly and had underlying medical conditions. All patients required ICU support and underwent various invasive procedures, including vascular access and tracheal intubation. Some patients also received invasive treatments and procedures, such as extracorporeal membrane oxygenation (ECMO) and intracranial surgery. The overall length of hospital stay was prolonged, with 66% of the patients either succumbing to their conditions or being discharged due to critical health status and economic constraints (Data S1 and S2). This suggests that patients co-infected with *A. baumannii* and *Elizabethkingia* experience a poor prognosis and a significant disease burden. Given that both strains were isolated from patient sputum samples, and antimicrobial susceptibility indicated that strains from different patients exhibited relatively consistent resistance phenotypes, suggesting high clinical homogeneity. To further investigate, we selected a patient (designated as P 04 in Data S1 and S2) with a poor clinical outcome despite aggressive treatment, from whom both *A. baumannii* strain AB2-1 and *E. anophelis* strain EA2-2 were isolated from the same sputum sample. These strains were obtained from a sputum culture collected on 21 May 2024, from a 58-year-old male patient in Nanchang, China. The patient had a history of diabetes and bioprosthetic aortic valve replacement. Upon admission, he was diagnosed with multiple organ dysfunction, respiratory failure, circulatory failure, severe pneumonia, pulmonary fungal infection, sepsis, and septic shock. In the ICU, he underwent tracheal intubation, extracorporeal membrane oxygenation (ECMO), and blood purification, receiving multiple antimicrobial treatments including piperacillin-tazobactam, meropenem, voriconazole, linezolid, azithromycin capsules, doxycycline, ampicillin, vancomycin, and polymyxin B. After 11 days of treatment, AB2-1 and EA2-2 were detected in the patient’s sputum. Despite aggressive medical interventions, the patient’s condition continued to deteriorate, and unfortunately, he passed away on the 13th day after AB2-1 and EA2-2 were isolated. We then conducted *in vitro* co-culture experiments on AB2-1 and EA2-2. Following DNA extraction and library construction from the two strains, we performed genome assembly and analysis using high-throughput sequencing to obtain the genomic information of AB2-1 and EA2-2.

### The genomes of *A. baumannii* AB2-1 and *E. anophelis* EA2-2

Bioinformatics analysis revealed that the AB2-1 genome comprises a 4.05 Mb chromosome, and the EA2-2 contains a 4.09 Mb chromosome, with no plasmids assembled in either strain (Table 3). Multilocus sequence typing (MLST) analysis indicated that AB2-1 belongs to the ST2 type, which is a predominant strain in hospital-acquired infections, whereas no ST type was detected for EA2-2.

**TABLE 3:** Genomic data for *A. baumannii* Ab2-1 and *E. anophelis* EA2-2

Strain	Genome size (bp)	GC (%)	No. gene	No. tRNA	No. rRNA
AB2-1	4,057,641	38.99	3939	73	18
EA2-2	4,090,918	35.69	3824	52	16

**Fig 1:**
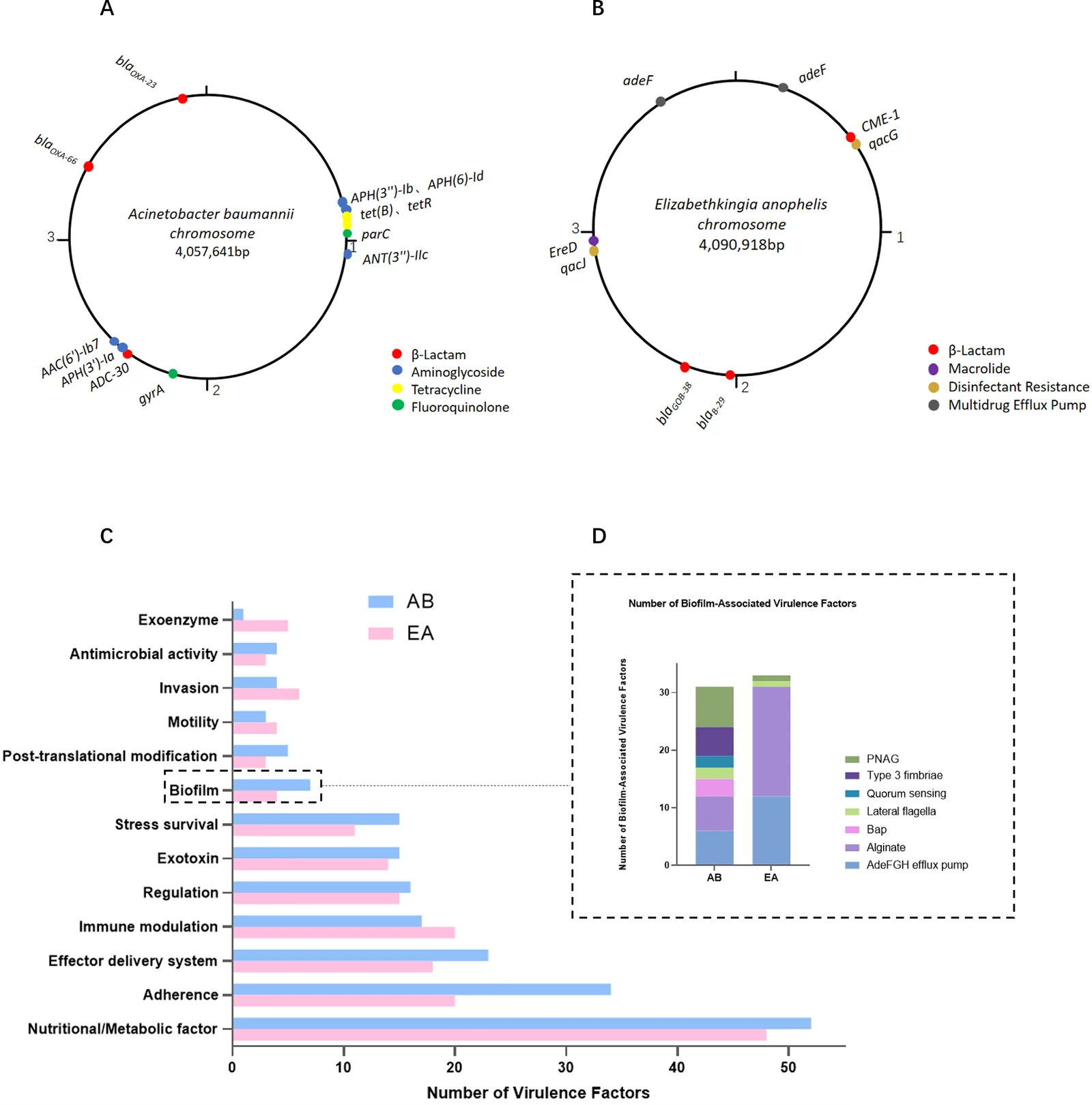
Genomic features of *A. baumannii* Ab2-1 and *E. anophelis* EA2-2. (**A**) Antibiotic resistance genes of AB2-1. (**B**) Antibiotic resistance genes of EA2-2. (**C**) Classification and counts of virulence factors in AB2-1 and EA2-2. (**D**) Biofilm-associated virulence factors and their counts in AB2-1 and EA2-2.

Since the two species, *A. baumannii* and *E. anophelis*, showed similar antibiotic resistance profiles, we annotated their genomic data using the CARD database (Data S3) to identify resistance determinants (Fig. 1A and B). Both strains are classified as carbapenem-resistant organisms (CROs), which is consistent with the data presented in Table 2. *Acinetobacter baumannii* and *Elizabethkingia* spp. isolated from 15 patients (including AB2-1 and EA2-2) exhibited a 100% resistance rate to carbapenem antibiotics. Among them, AB2-1 harbors the *bla_OXA-23_* and *bla_OXA-66_* genes, which encode carbapenemases capable of hydrolyzing the β-lactam ring of carbapenem antibiotics, thereby conferring resistance (28). In contrast, strain EA2-2 possesses the *bla_B-29_* and *bla_GOB-38_* genes, which encode metallo-β-lactamases (MBLs) that confer resistance to most β-lactam (11, 29). In addition to its resistance to carbapenems, AB2-1 also exhibits resistance to aminoglycosides and fluoroquinolones. Furthermore, the efflux pump system of AB2-1 is highly active and encompasses multiple drug efflux pump-related genes (30) and could lead to multidrug resistance (MDR). In comparison, while EA2-2 demonstrates strong carbapenem resistance, it carries fewer resistance genes, resulting in a more concentrated resistance spectrum. Notably, the *qacG/qacJ* gene has been identified in EA2-2, which encodes a disinfectant resistance protein that can tolerate 0.1% benzalkonium chloride, contributing to its prolonged survival in hospital environments (31).

In-depth exploration of the virulence mechanisms of AB2-1 and AB2-2 involved annotating the complete genome sequences of the bacteria using the VFDB database (Data S4), with the primary virulence mechanisms illustrated in Fig. 1C. In the functional classification of virulence factors, both strains exhibited significantly higher counts in the categories of nutritional/metabolic factor, immune modulation, effector delivery system, and adherence compared with other categories, indicating strong environmental adaptability, immune modulation, and pathogenic capabilities of the two strains. Among these, the abundance of nutritional/metabolic factors is the highest, reflecting that both may employ multiple strategies to acquire nutrients within the host to sustain the metabolic activities required for their survival. Notably, the nutritional/metabolic factors of AB2-1 include various iron uptake systems, which can enhance the strain’s survival capability in the host’s low-iron environment. Studies have shown that iron concentration can influence bacterial motility by regulating the type I and type IV pilus systems. In environments with low iron concentrations, 18% of motility-related genes (many of which are associated with the type I and type IV pilus systems) are significantly downregulated (32), which is closely related to the colonization and persistent infection of *A. baumannii* (33, 34). Beyond such regulatory mechanisms, adhesion factors themselves play a crucial role in bacterial colonization and persistent infection. The adhesion factors of both strains involve various structures such as flagella, pili, and membrane proteins, which aid in their colonization on host surfaces and biofilm formation, constituting a key step in initiating infection (35–37). In comparison, AB2-1 possesses a greater variety and number of adhesion factors, particularly a significant increase in genes related to type IV pili, as well as multiple pilus systems associated with surface attachment and biofilm formation, such as AF/R1 pili and MR/P fimbriae (38, 39). In contrast, EA2-2 has relatively fewer adhesion factors, primarily consisting of non-specific adhesion proteins like EF-Tu and Hsp60 (40, 41), and shows a relative deficiency in pilus systems. This difference suggests that AB2-1 may possess stronger colonization capacity and competitive advantage.

The production of biofilms facilitates a series of complex intra- and inter-species interactions among microorganisms, enabling the cross-talk of signaling pathways (42) and the exchange of genetic materials and nutrients through cross-feeding, which is of significant importance for microbial adaptability and virulence (43–45). We conducted a detailed analysis of the biofilm-related virulence factors of these two strains (Fig. 1D). AB2-1 contains seven categories of biofilm-related virulence factors, which are involved in polysaccharide matrix synthesis (46, 47), intercellular adhesion, surface attachment (35, 48), and quorum sensing signaling (30, 49), collectively promoting biofilm formation. The combined effects of multiple factors enable it to stably survive within the host and on the surfaces of medical devices, which is one of the mechanisms contributing to the widespread prevalence of *A. baumannii* in hospital environments (50). In contrast, EA2-2 was detected with only four types of virulence factors at the same level, lacking Bap protein, Type 3 fimbriae, and the structure of the quorum sensing system, suggesting that its biofilm formation capability primarily relies on the synthesis of extracellular polysaccharides and is relatively weaker in terms of adhesion and functional regulation. The biofilms it forms may be more susceptible to immune clearance or destruction by antimicrobial drugs (35, 46–48). This difference may, to some extent, explain the phenomenon observed in this study that the biofilm formation capability of AB2-1 is stronger than that of EA2-2 (see below), suggesting that under the same context, *A. baumannii* has a greater potential for persistent infection.

### Growth and *in vitro* Cross-Protection between AB2-1 and EA2-2

Since both strains in this study exhibited similar resistance to most antimicrobial agents, we employed the microbroth dilution method to further determine the minimum inhibitory concentration (MIC) of AB2-1, EA2-2, and their 1:1 co-culture against common antibiotics, including piperacillin (penicillins), cefotaxime (cephalosporins), gentamicin (aminoglycosides), meropenem (carbapenems), and doxycycline (tetracyclines). The results indicated that AB2-1 was highly resistant to piperacillin sodium, cefotaxime, and meropenem, and also resistant to gentamicin and doxycycline; EA2-2 was sensitive to doxycycline but highly resistant to the other antibiotics (Table 4).

**TABLE 4:** Minimum inhibitory concentrations for mono-cultures and co-cultures (1:1) of AB2-1 and EA2-2 (μg/mL)

Strain	Piperacillin	Cefotaxime	Gentamicin	Meropenem	Doxycycline
AB2-1	256	256	16	128	128
EA2-2	256	256	256	256	4
AB + EA (1:1)^*a*^	256	256	256	256	128

^*a*^ AB + EA = AB2-1 + EA2-2.

**Fig 2:**
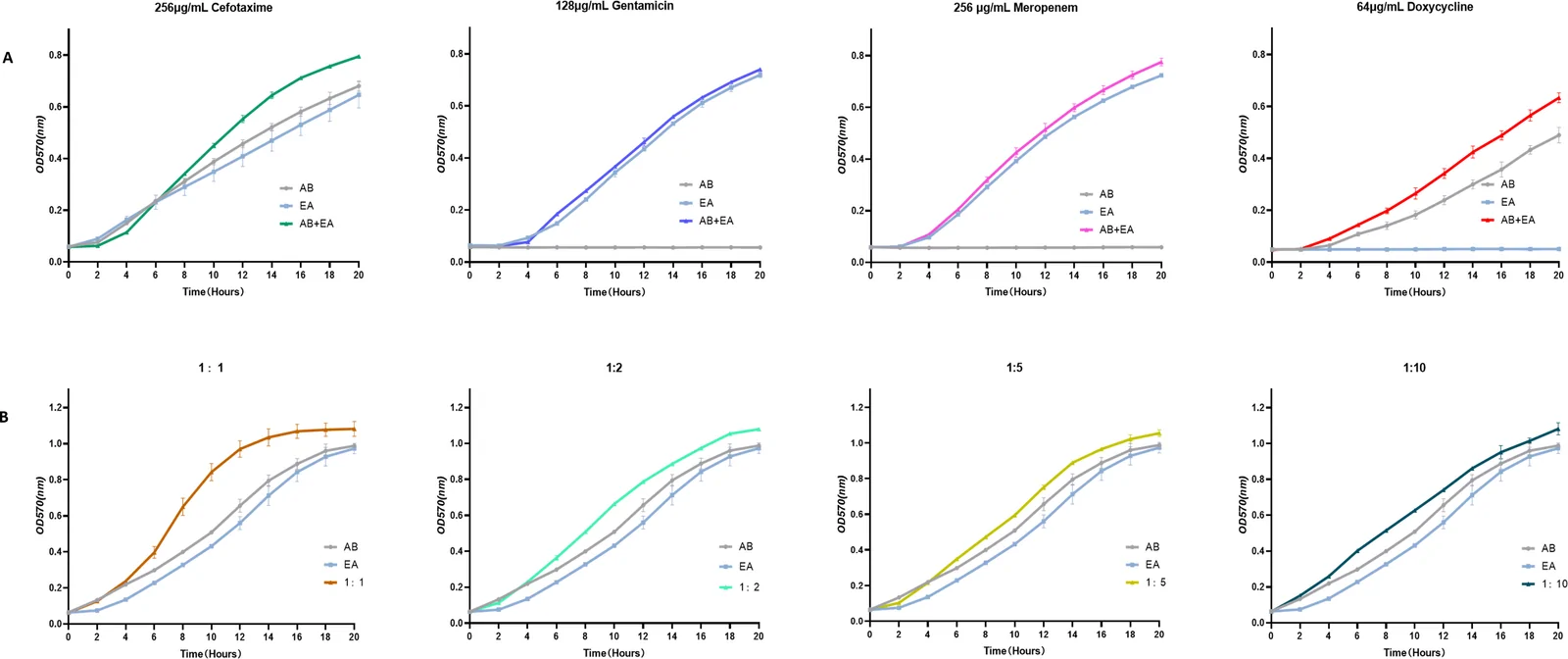
Growth of AB2-1 and EA2-2. (**A**) Growth curves of mono-cultures and co-cultures under different antibiotic treatments. AB and EA were co-cultured at a 1:1 ratio. (**B**) Growth of mono-cultures and co-cultures at different mixing ratios. Ratios of 1:1, 1:2, 1:5, and 1:10 refer to AB:EA.

Given the significant difference in susceptibility to doxycycline between these two strains (AB2-1 resistant, EA2-2 sensitive), we performed co-culture growth assays under planktonic conditions in the presence of 64 μg/mL doxycycline. This concentration is much higher than the MIC of EA2-2, yet not sufficient to completely kill AB2-1. Under these conditions, the 20-h growth curves showed that the single strain EA2-2 did not grow at all. Exponential-phase growth rates were calculated by linear regression of log-transformed OD values between 4 and 12 h. Under doxycycline exposure, the growth rate of Ab2-1 in co-culture with EA2-2 (0.178 ± 0.004 h^−^¹) was higher than that observed in monoculture (0.136 ± 0.005 h^−^¹) (unpaired *t*-test, *P* < 0.001) (Fig. 2A).

A similar phenomenon was observed under conditions where both parties exhibited resistance to antibiotics. In the growth curves plotted for treatments with cefotaxime (256 μg/mL), gentamicin (128 μg/mL), and meropenem (256 μg/mL), the maximum biomass (OD570) of the co-culture exposed to cefotaxime remained higher than that of the monoculture, which is likely attributable, at least in part, to the degradation of the antibiotic facilitated by β-lactamases produced by both strains. However, the maximum biomass of the co-cultures treated with gentamicin and meropenem was not statistically significant compared with that of the monocultures (Fig. 2A). Subsequently, the co-cultures treated with doxycycline, gentamicin, and meropenem were plated and incubated at 37°C for 24 h. On the plates treated with doxycycline, two types of colonies of varying sizes were observed. Mass spectrometry identification confirmed these as *A. baumannii* and *E. anophelis*. In contrast, gentamicin- and meropenem-treated plates yielded colonies of a single morphology, indicating the survival of only one species (Fig. S1). Taken together, these results indicate that cross-protection in co-culture occurs only under antibiotic conditions in which there is a pronounced difference in susceptibility between the two strains. Specifically, under doxycycline treatment, AB2-1 protected EA2-2 from antibiotic exposure, whereas no cross-protection was observed under gentamicin or meropenem treatment. These findings suggest that, in co-culture, when a marked difference in antibiotic resistance exists, AB2-1 may mitigate the inhibitory effects of antibiotics on EA2-2 through an interspecies interaction–dependent mechanism.

In addition, in growth kinetic experiments conducted without drug pressure, logarithmic-phase cultures of the two strains were adjusted to a McFarland turbidity of 0.5, mixed at different ratios, and used to plot 20-h growth curves. The results showed that co-cultures reached a higher maximum biomass (OD value) in the stationary phase compared to monocultures (Fig. 2B). Specifically, when co-cultured at a 1:1 inoculation ratio, the exponential-phase growth rate (0.178 ± 0.004 h^−^¹) was significantly higher than that of AB2-1 in monoculture (0.136 ± 0.005 h^−^¹) (unpaired *t*-test, *P* < 0.001).

### Transcriptomic characteristics of AB2-1 and EA2-2

**Fig 3:**
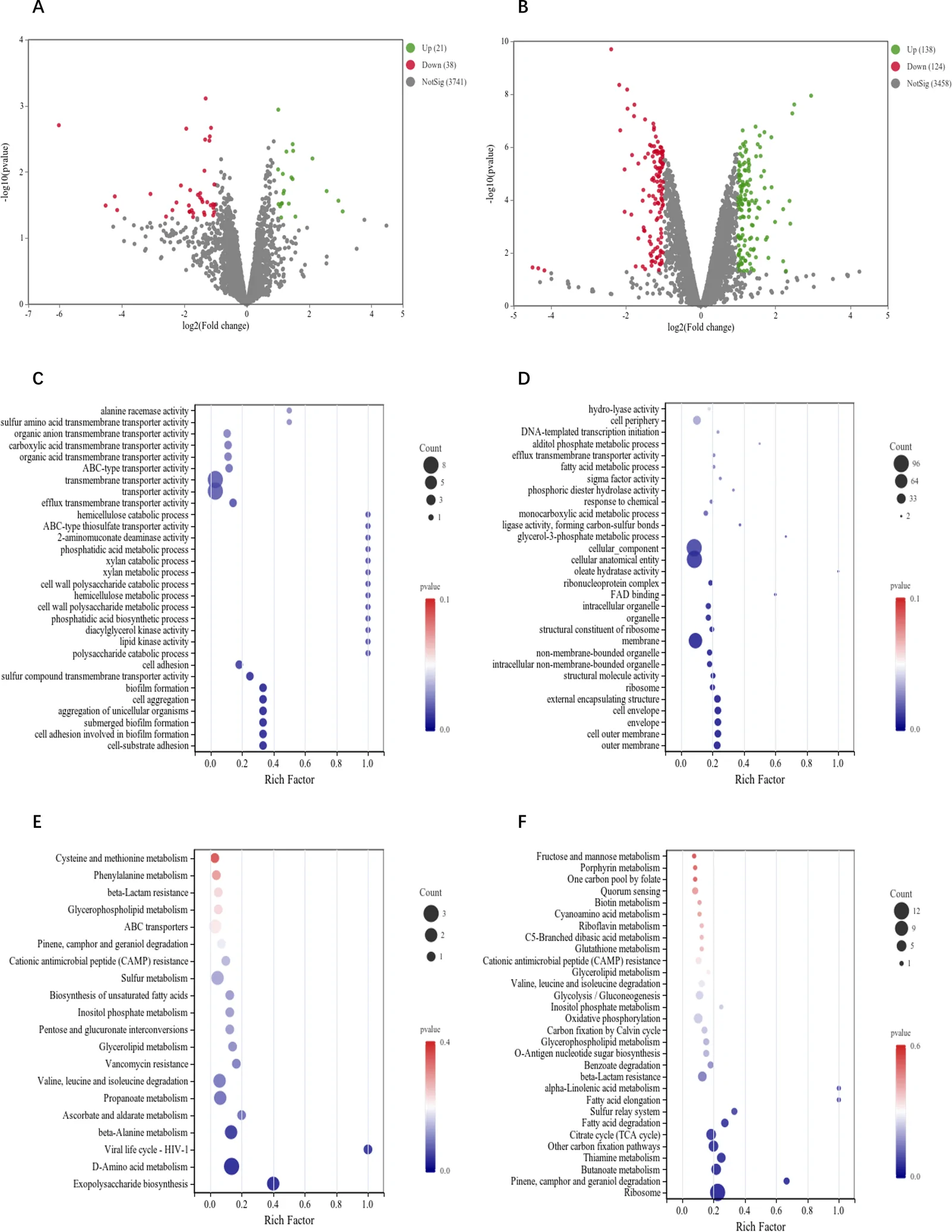
Transcriptomic features of AB2-1 and EA2-2 following co-culture. (**A and B**) Gene expression changes in AB2-1 (**A**) and EA2-2 (**B**) after co-culture compared with their respective monocultures. (**C and D**) GO (**C**) and KEGG (**D**) enrichment analyses of AB2-1 after co-culture relative to monoculture. (**E and F**) GO (**E**) and KEGG (**F**) enrichment analyses of EA2-2 after co-culture relative to monoculture.

To elucidate the mechanisms underlying the cross-protection between AB2-1 and EA2-2 in co-culture, transcriptomic sequencing was performed on both monocultured and co-cultured AB2-1 and EA2-2 under biofilm conditions. Total RNA was extracted using the Trizol method, and strand-specific libraries were constructed, followed by bioinformatics analysis of the obtained raw sequencing data. Compared with monoculture, a total of 321 differentially expressed genes (DEGs) (*P*-value < 0.05, |log2FC| > 1) were identified after co-culture, with 159 genes significantly upregulated and 162 genes significantly downregulated (Fig. 3A and B). The number of DEGs in EA2-2 was greater than that in AB2-1, indicating that co-culture had a more pronounced effect on EA2-2. Among the DEGs, AB2-1 involved upregulated genes related to cell membrane formation, cell wall, amino acid metabolism, lipid metabolism, and transcriptional regulation, while EA2-2 involved upregulated genes related to transmembrane transport, oxidative stress, cofactor synthesis, and signal transduction. Additionally, we conducted enrichment analysis on the expression pathways of AB2-1 and EA2-2 after co-culture (Fig. 3C through F).

**Fig 4:**
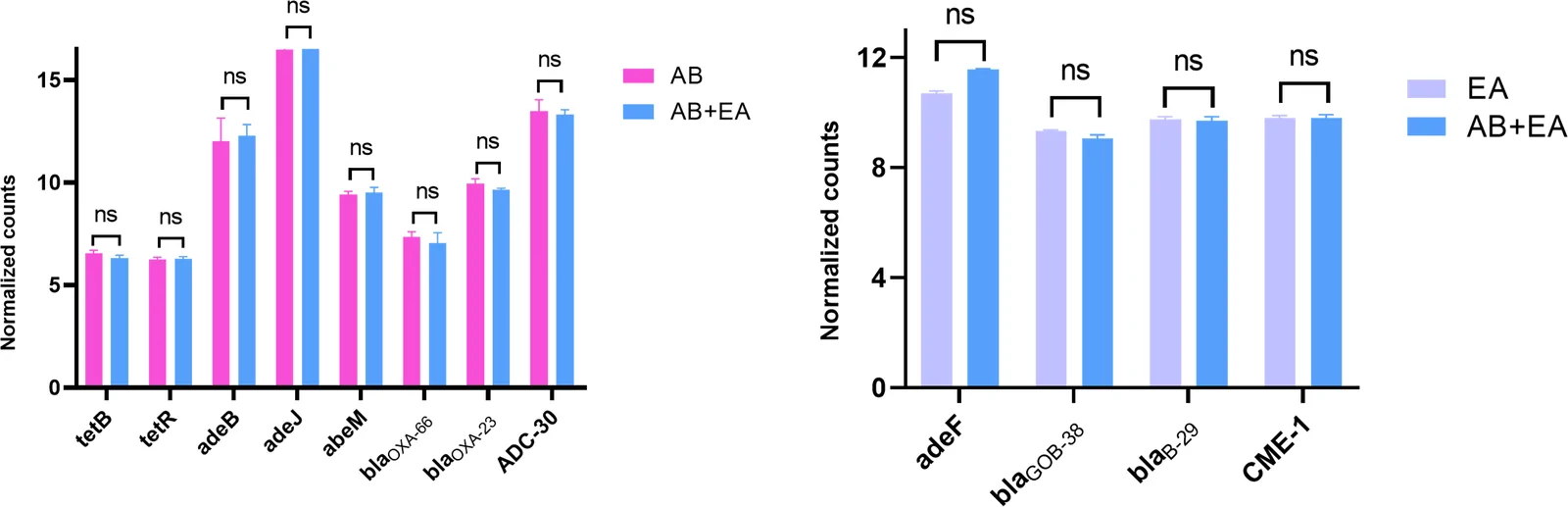
Transcriptomic levels of tetracycline-related, selected efflux pump, and major resistance genes in AB2-1 and EA2-2 following co-culture. Data are expressed as the mean and standard error of three independent biological replicates (ns, not significant).

Genomic analysis under monoculture conditions revealed that AB2-1 harbors the *tetB* and *tetR* genes (Fig. 1A), which are closely associated with bacterial resistance to tetracycline-class antibiotics (51, 52). By comparison, no tetracycline-related resistance determinants were identified in the genome of EA2-2 (Fig. 1B; Data S3), indicating a genetic basis for differential susceptibility to tetracyclines between the two strains. However, under co-culture conditions, EA2-2—originally sensitive to doxycycline—was able to survive at a concentration 16 times its MIC, suggesting an altered phenotypic susceptibility. Transcriptomic analysis further revealed that following co-culture, the expression of tetracycline resistance-associated genes in AB2-1, including *tetB*, *tetR*, and the classical efflux pump genes *adeB*, *adeJ*, and *abeM*, was not significantly upregulated. Similarly, no tetracycline resistance genes were detected in EA2-2, and its efflux pump gene *adeF* showed no significant change in expression. Moreover, the major resistance genes of both strains—including *bla_OXA-66_*, *bla_OXA-23_*, and *ADC-30* in AB2-1, and *bla_GOB-38_*, *bla_B-29_*, and *CME-1* in EA2-2—were not transcriptionally altered after co-culture (Fig. 4). KEGG enrichment analysis further indicated that no classic resistance-related pathways, such as “β-lactam resistance” or “ABC transporters,” were significantly enriched in EA2-2. Instead, pathways related to structural adhesion, signal response, and quorum sensing were predominantly enriched (Fig. 3F). These findings collectively suggest that EA2-2 does not appear to acquire doxycycline resistance independently after co-culture. Rather, it likely depends on external environmental changes or protective effects provided by AB2-1, jointly enhancing resistance to doxycycline.

### Co-cultivation enhances biofilm formation of AB2-1 and EA2-2

Biofilm formation is widely recognized as a crucial factor for microbial survival in both natural and clinical environments. It serves as a medium for interspecies resource allocation and information transfer and is also an important mechanism of antibiotic resistance (42, 53). For instance, Semenec et al. demonstrated that in the biofilm context, *A. baumannii* confers antibiotic protection to *Klebsiella pneumoniae* through the secretion of cephalosporinase during co-culture (54). In our study, since the drug resistance genes of the two strains did not show significant upregulation after co-culture, and the biofilm formation-related pathways were enriched in AB2-1, we further explored whether co-culture enhances drug resistance by increasing biofilm formation, thereby through physical barriers or interactions between the strains.

**Fig 5:**
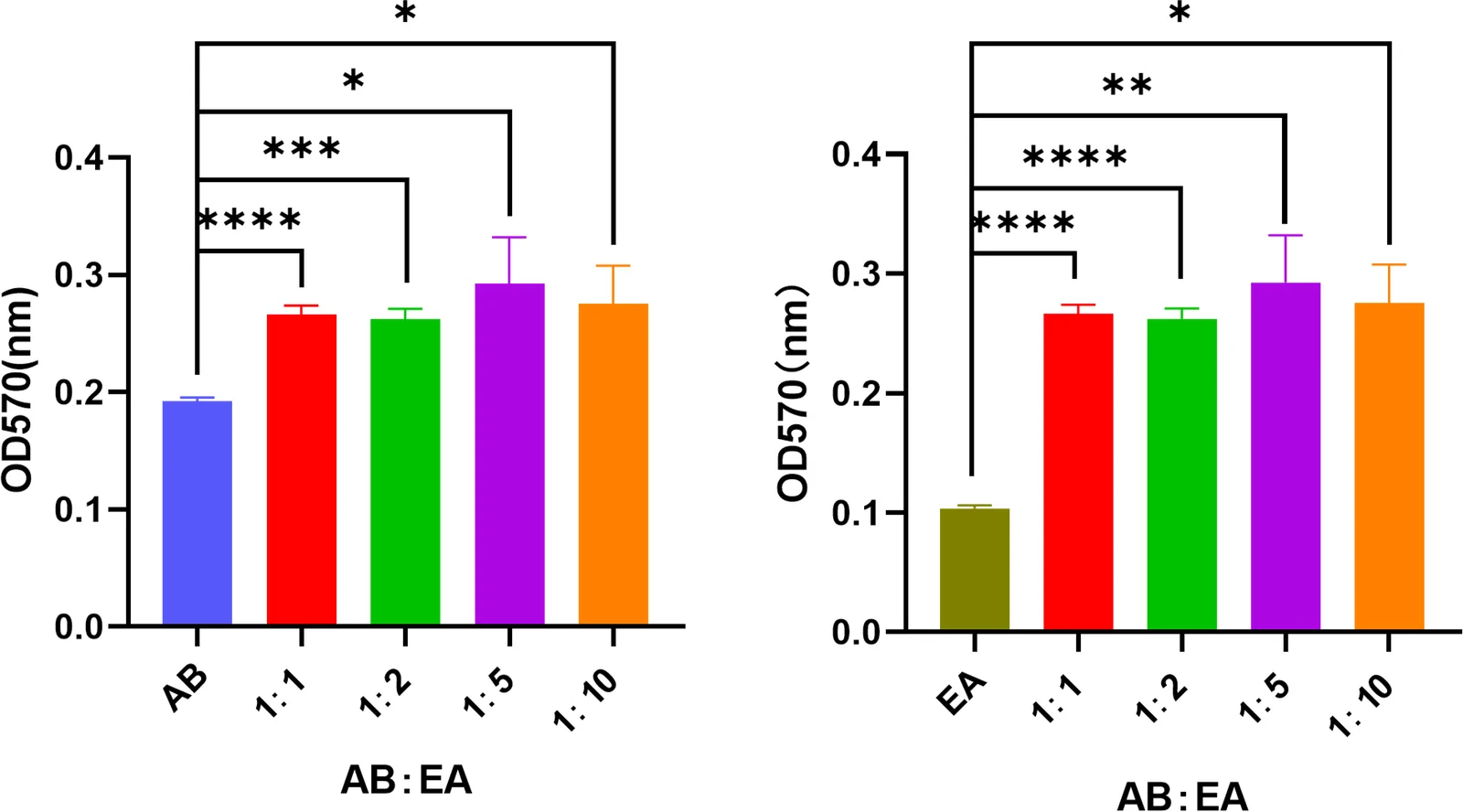
Comparison of biofilm formation between mono-cultures and co-cultures at different mixing ratios. Ratios of 1:1, 1:2, 1:5, and 1:10 refer to AB:EA. Data are expressed as the mean and standard error of three independent biological replicates. (*P < 0.05; **P < 0.01; ***P < 0.001; and ****P < 0.0001).

**Fig 6:**
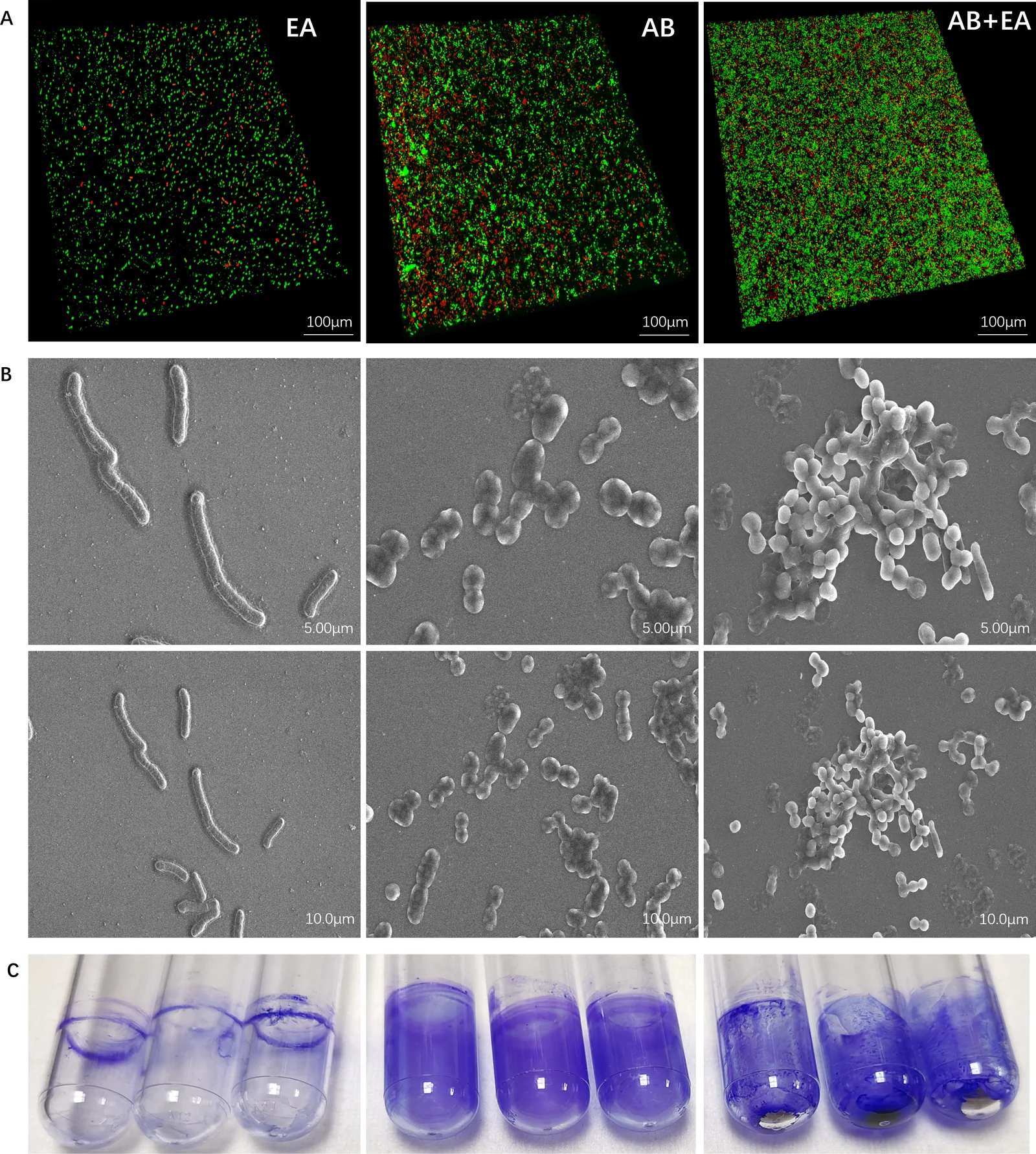
Characteristics of biofilms formed by AB2-1 and EA2-2 in mono- and co-cultures. (**A**) CLSM images of biofilms grown in small test tubes, stained with the LIVE/DEAD Bacterial Staining Kit with DMAO & PI; live cells are shown in green and dead cells in red. (**B**) SEM images of biofilms under mono- and co-culture conditions at two different magnifications. (**C**) Crystal violet staining of biofilms after 3 days of mono- and co-culture. AB, *A. baumannii* AB2-1 mono-culture; EA, *E. anophelis* EA2-2 mono-culture; AB+EA, co-culture of both strains.

First, the amount of biofilm formation was detected using crystal violet staining. The amount of biofilm formation under different mixing ratios was higher than that under single culture (Fig. 5), indicating that the two strains may construct a more complex biofilm structure through synergistic effects, which may be closely related to their persistent colonization and enhanced drug resistance in mixed infections. By visual observation of the biofilm on the culture tubes, the biofilm of AB2-1 was more apparent than that of EA2-2 after 3 days of single culture (Fig. 6C). Next, the LIVE/DEAD Bacterial Staining Kit with DMAO & PI was used to perform live/dead staining on the mono- and co-cultured biofilms, which were further observed under a confocal laser scanning microscope (CLSM). The biofilm formed by EA2-2 in monoculture was relatively thin with a sparse structure and a higher proportion of live cells. In contrast, AB2-1 in monoculture formed a biofilm of moderate thickness, with a similar proportion of live and dead cells. However, during co-culture, the biofilms formed by both strains were thicker, more densely structured, and exhibited a higher proportion of live cells (Fig. 6A).

To further elucidate the morphological structure of the biofilm post-co-culturing, the biofilms from mono- and co-cultures were observed under scanning electron microscopy (SEM). The mono-cultured AB2-1 exhibited a loosely aggregated spherical form, while EA2-2 displayed a dispersed distribution of elongated rods. Under co-culturing conditions, the two strains adhered closely, forming aggregated structures (Fig. 6B). These results indicate that co-culturing enhances the overall ability to form biofilms. The dense biofilm structure may slow the diffusion of antibiotics to the bacterial community, providing biofilm protection for *Elizabethkingia*. The cellular environment within the biofilm may facilitate interspecies signaling, enabling the exchange or sharing of metabolic substances. Additionally, co-culturing may influence bacterial viability.

**Fig 7:**
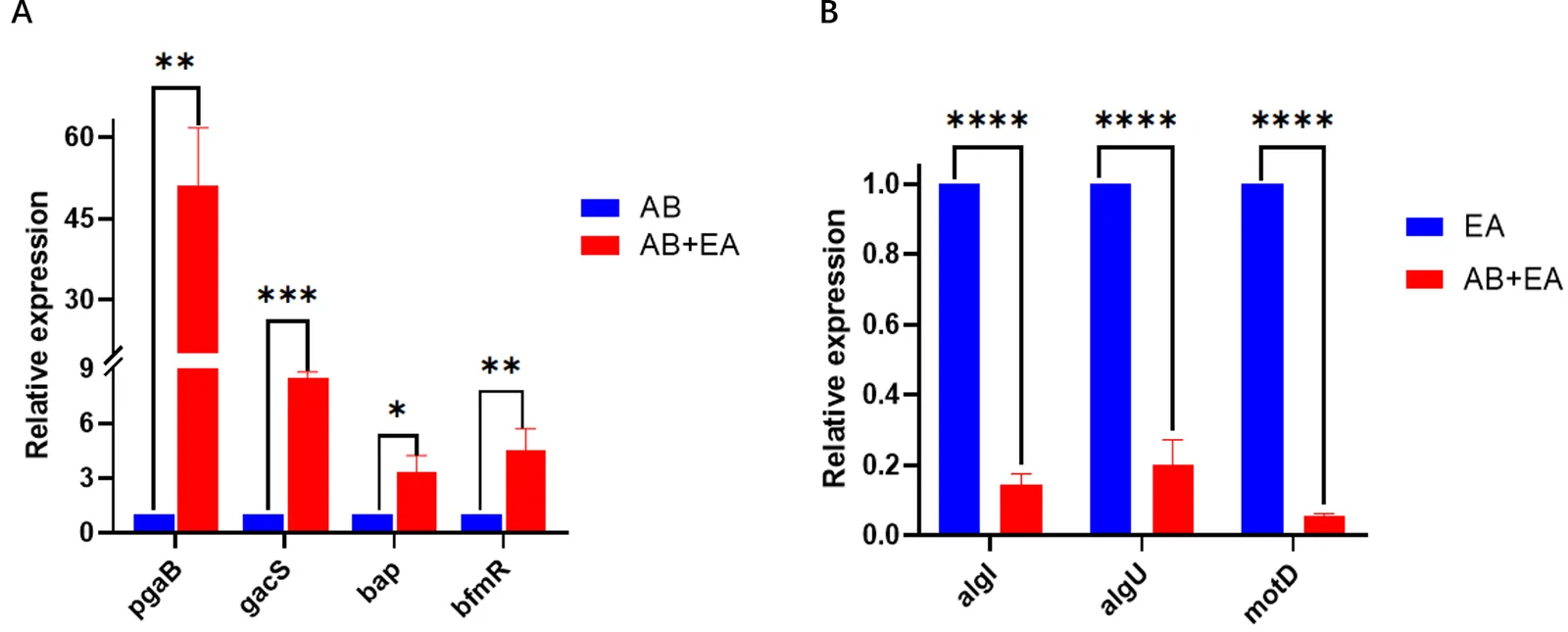
Relative expression levels of biofilm-associated genes in AB2-1 (**A**) and EA2-2 (**B**) after co-culture. AB and EA were co-cultured at a 1:1 ratio. Data are expressed as the mean and standard error of three independent biological replicates. (*P < 0.05; **P < 0.01; ***P < 0.001; and ****P < 0.0001).

Transcriptomic analysis after co-culture further revealed that in AB2-1, both the Poly-beta-1,6-N-acetyl-D-glucosamine synthase and Poly-beta-1,6-N-acetyl-D-glucosamine N-deacetylase encoded by the *pgaB* gene were upregulated. These two enzymes collectively promote the synthesis and modification of PNAG (55–58), which is one of the main components of the extracellular polymeric substance (EPS). As a widely present biofilm component in microorganisms, PNAG mediates numerous virulence traits, including host colonization and antibiotic resistance (47, 59, 60). The increased expression of the *pgaB* gene can enhance the stability and pathogenicity of biofilms, potentially improving the bacteria’s barrier effect against antibiotics. Further validation using RT-qPCR on biofilm formation-related genes in both bacterial strains was consistent with the transcriptomic results (Fig. 7). The relative expression level of the *pgaB* gene in AB2-1 was significantly higher in co-culture compared with monoculture, while the relative expression levels of biofilm-related genes in EA2-2 decreased. This indicates that in the co-culture system, the biofilm formation ability of EA2-2 was not significantly enhanced, whereas AB2-1 played a major role in promoting overall biofilm formation, potentially serving as a key factor in the structural and functional characteristics of the biofilm post co-culture.

### Co-infection with AB2-1 and EA2-2 enhances virulence towards the host

**Fig 8:**
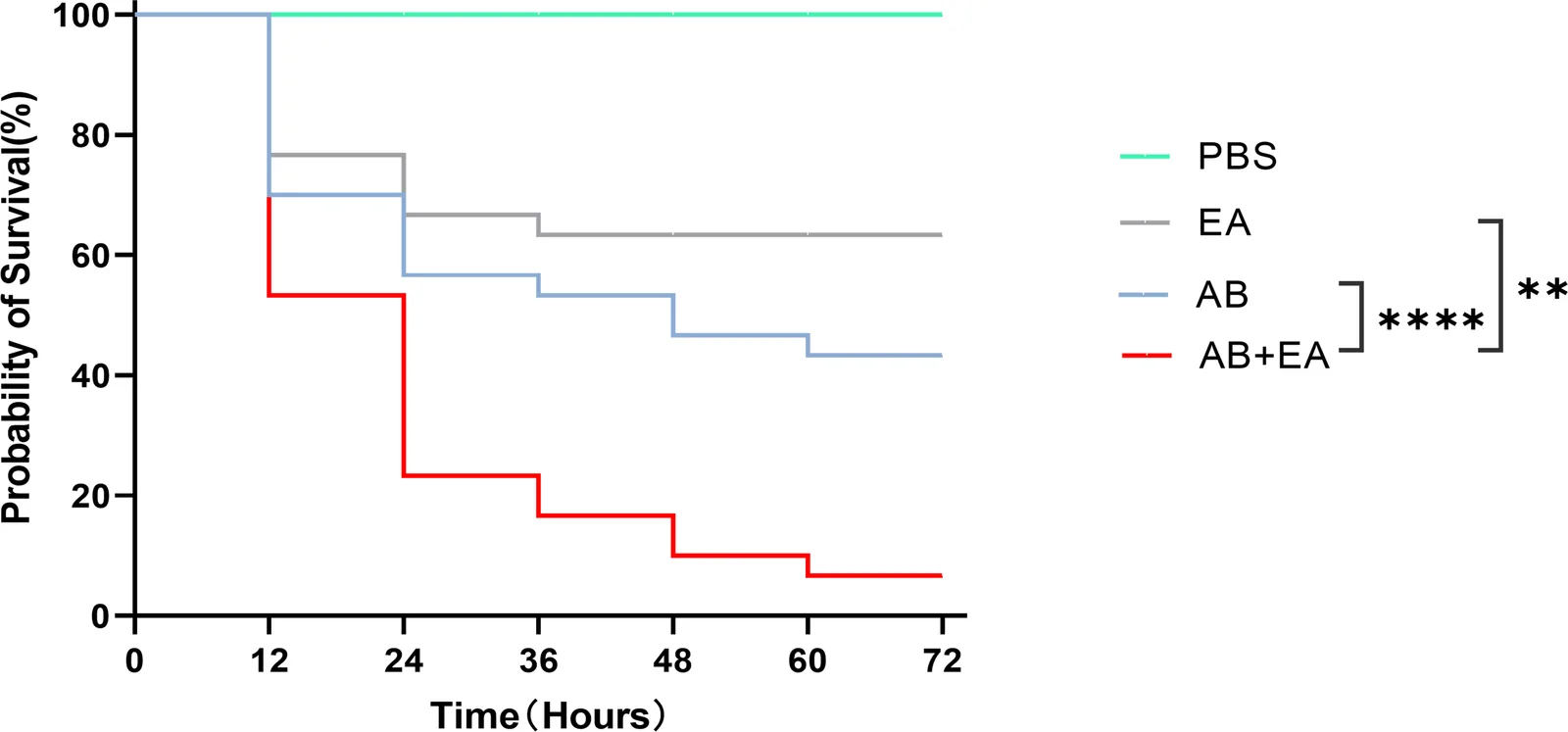
Virulence of AB2-1 and EA2-2 in *Galleria mellonella*. AB+EA: 1:1 co-infection of AB2-1 and EA2-2. Three biological replicates were included for each group. PBS only controls had 100% survival (**P < 0.01 and ****P < 0.0001).

To compare the virulence differences between AB2-1 and EA2-2 during single and co-infection, an infection model was established using *Galleria mellonella* larvae. The bacterial suspensions for single and co-culture were adjusted to 10^7^ CFU/mL, and each larva was injected with 10 μL. The larvae were then incubated in the dark at 37°C for 72 h, with survival rates recorded every 12 h to plot survival curves (Fig. 8). The results showed that virulence levels were enhanced during co-infection, killing 93% of the larvae within 60 h, while the virulence of AB2-1 alone was second, with a mortality rate of 56% within 72 h. The virulence of EA2-2 was the lowest, with a larval survival rate of 63% after 72 h. This indicates that co-infection with both bacteria significantly increases the virulence level compared with single infection, suggesting a certain synergistic effect between the two bacteria that enhances their infectious capability.

## DISCUSSION

Clinically common nosocomial infections are often mixed infections, which are prevalent in cases, such as ventilator-associated pneumonia (23), catheter-related bloodstream infections (61, 62), and chronic wound infections (63). These infections frequently lead to the failure of antimicrobial therapy and exacerbate disease progression (22, 64), thereby increasing the treatment burden (65). This study found that co-infections of *A. baumannii* AB2-1 and *E. anophelis* EA2-2 can occur in patients admitted to the ICU, those with underlying diseases, and those undergoing invasive procedures, such as endotracheal intubation, increasing the difficulty of treatment. *In vitro* co-culture experiments demonstrated that EA2-2 exhibited enhanced resistance to doxycycline after co-culture. Biofilm phenotype and transcriptome analysis further indicated that the two bacteria could enhance biofilm formation during co-culture, with the *pgaB* gene of AB2-1 potentially playing a key role.

Microbial communities in the environment often form complex interactive networks, exhibiting intricate, multi-species metabolic interactions (66). The mechanisms of microbial interactions include signal transduction, cross-feeding, and biofilm protection, among others (54, 67, 68). In the context of biofilms, co-culturing AB2-1 can protect EA2-2 from doxycycline killing at concentrations 16 times higher than its own MIC, which is consistent with the report by Smith et al., where the bactericidal activity of meropenem against *S. aureus* was reduced when co-cultured with carbapenem-resistant *A. baumannii* (69). Liao et al. also found that in response to antibiotics, some community members can activate the tolerance of others to achieve cross-protection (70). After co-culturing, the major resistance genes of the two strains were not significantly upregulated, while the expression of *pgaB* in AB2-1 was enhanced, potentially leading to a richer and more densely structured biofilm. It is speculated that the biofilm dominated by AB2-1 is enhanced after co-culturing, providing cross-protection for EA2-2. The synergistic effect of the two may alter the population’s antibiotic resistance, thereby forming a symbiotic system with cross-protection capabilities.

Transcriptome analysis also revealed that the efflux pump system may be involved in drug resistance. Efflux pumps are a mechanism by which bacteria interact with their environment, protecting them from toxic compounds, antimicrobial molecules, reactive oxygen species (ROS), and toxic byproducts of biochemical degradation pathways produced by other species of bacteria or the host (71). For instance, MdfA is a classic Major Facilitator Superfamily (MFS) multidrug transporter that can expel a variety of broad-spectrum antibiotics out of the cell (72), potentially providing protection to neighboring bacteria. Notably, under co-culture conditions, although no significant upregulation of tetracycline-associated efflux pumps was detected in either strain, the expression levels of several efflux pump-related genes—including *mexA* (log2FC = +1.276), *bepE* (log2FC = +1.034), *bepF* (log2FC = +1.131), and *oprM* (log2FC = +1.126)—were elevated in EA2-2. These systems are known to export a broad range of antimicrobial substances or toxic compounds and may therefore contribute to enhanced general stress tolerance in a mixed-species environment (61, 63, 64). In addition, the expression of the cell wall acetylase gene *oatA* (AB2-1:log2FC =+1.156, EA2-2: log2FC = +1.312) was also elevated in both strains after co-culture. It has been reported that OatA-mediated peptidoglycan modification can reduce lysozyme binding and enhance cell envelope robustness, which may indirectly improve bacterial survival under antimicrobial pressure (73, 74). GO enrichment analysis revealed significant enrichment of AB2-1 in biofilm formation, cell adhesion, and cell wall polysaccharide metabolic process. KEGG analysis also indicated an enhancement in the exopolysaccharide biosynthesis pathway, consistent with the upregulation of PNAG synthase and PNAG N-deacetylase. In contrast, the EA2-2 quorum sensing (QS) pathway was found to be enriched (Fig. 3). QS can regulate intercellular communication, the secretion of public goods, and the synthesis of antimicrobial substances, thereby enhancing its adaptability to the environment and antibiotics (18, 75). Specifically, QS is an important mechanism by which Gram-negative bacteria regulate collective behaviors and interspecies interactions. Through QS signal molecules, bacteria can coordinate multiple physiological processes, including growth, biofilm formation, virulence factor expression, transcriptional regulation, and metabolic cross-feeding (76). Previous studies have demonstrated that QS participates in regulating interspecies interactions in various co-culture systems. For example, in co-culture studies of *P. aeruginosa* and *S. aureus*, proteases and exotoxins secreted by *P. aeruginosa* have been shown to be closely associated with the QS signal molecule AI-2 produced by *S. aureus* (77). In a co-culture model of *Lactobacillus plantarum* and *Saccharomyces cerevisiae*, transcriptomic analysis revealed upregulation of QS-related genes in *L. plantarum*, whereas genes associated with growth were downregulated in *S. cerevisiae*, suggesting that QS may be involved in regulating cross-species interactions (78). In the present study, significant enrichment of the QS pathway was observed in EA2-2 under co-culture conditions, suggesting that QS may play a regulatory role in the interaction between AB2-1 and EA2-2, potentially influencing biofilm-related characteristics, stress adaptation, or metabolic cooperation. However, the specific QS signals and regulatory mechanisms involved remain to be further elucidated in future studies.

Additionally, the two strains may also dynamically regulate survival resources under co-culture conditions, which could facilitate coexistence and synergism. After co-cultivation, multiple iron metabolism-related genes in AB2-1 were upregulated, indicating enhanced iron uptake and utilization. In contrast, EA2-2 upregulated iron receptor genes, including the TonB system, to cope with iron limitation stress (79, 80). Additionally, EA2-2 actively responded to survival resource pressures by upregulating various genes related to oxidative stress (*MsrB* and *RclC*) (81, 82), cell wall/membrane stress (*OatA*, *MepM,* and *SigG*) (73, 83, 84), and nutritional starvation (*GlpD/F/K*) (85–87), which facilitates its coexistence with AB2-1 and may help maintain microbial community stability.

This study revealed through phenotypic experiments, genomic and transcriptomic analyses that AB2-1 primarily provided cross-protection to EA2-2 by enhancing biofilm formation during co-culture, suggesting a synergistic mechanism between the two, providing a basis for understanding the interactions in polymicrobial infections and offering new insights and directions for developing more effective treatments for polymicrobial infections. However, this study still has limitations: the limited number of strains included may not fully reflect the interaction patterns between *A. baumannii* and *Elizabethkingia* across different hosts; the transcriptomic analysis was based solely on the logarithmic growth phase, failing to capture the entire process. Future research could expand the sample size of clinical isolates and incorporate time-series transcriptomics to systematically investigate the dynamic regulatory mechanisms of interspecies interactions.

## MATERIALS AND METHODS

### Clinical data collection

The clinical information of patients in this study was obtained from the First Affiliated Hospital of Nanchang University in Jiangxi Province, China. *A. baumannii* and *Elizabethkingia* were isolated from the patients’ sputum and identified through matrix-assisted laser desorption/ionization time-of-flight mass spectrometry (Bruker Daltonics). The antimicrobial susceptibility assays were carried out using the VITEK II system (BioMerieux, France), and the evaluation of susceptibility outcomes was performed following the guidelines established by the Clinical and Laboratory Standards Institute (CLSI). The selected antibiotics constitute the standardized antimicrobial susceptibility testing panel routinely used in our hospital for Gram-negative clinical isolates. This panel covers the major antibiotic classes commonly employed in the treatment of nosocomial infections. This study involving clinical data were reviewed and approved by the Institutional Review Board (approval no. IIT2024667).

### Bacterial strains and growth conditions

*A. baumannii* AB2-1 and *E. anophelis* EA2-2 were isolated from the same sputum specimen of a patient with pneumonia and were selected as a representative strain pair for subsequent *in vitro* co-culture experiments. This selection was based on the confirmed co-isolation from a single clinical specimen and the observation that their antimicrobial resistance profiles were consistent with those of the majority of co-infected patients included in this study (Table 2). In all experiments, bacteria were cultured overnight at 37°C and harvested during the logarithmic growth phase after shaking at 180 rpm. Unless otherwise stated, all co-culture experiments were conducted at a 1:1 ratio.

### Antimicrobial susceptibility testing and cross-protection experiments

Strains AB2-1 and EA2-2 were streaked onto Mueller Hinton agar (MH agar) plates and placed in an incubator overnight at 37°C. A solitary colony was then selected, suspended in sterile saline, and standardized to match a 0.5 McFarland turbidity standard. This suspension was subsequently diluted 100 times in cation-adjusted Mueller Hinton broth (CAMH, Solarbio) to generate a mixed culture of AB2-1 and EA2-2 in a 1:1 ratio. After gradient dilution of various antibiotics, 100 μL of either the individual culture or the mixed culture was introduced into a 96-well plate, which contained 100 μL of antibiotics at various concentrations. The plates were incubated for 16 to 20 h at 37°C, and the results were interpreted using the CLSI breakpoint (34th edition, 2024) to assess the MIC values of the single and co-cultures. Based on the MIC results, concentrations between the MICs of the two strains were selected for antibiotics with significant susceptibility differences, and for antibiotics where both strains exhibited high resistance, concentrations close to the MIC were chosen for 24-h treatment. Following this, the co-cultures were diluted and evenly spread onto blood agar plates, which were incubated at 37°C for 24 to 28 h to observe colony growth. The antibiotics used in this study were purchased from the following commercial suppliers: cefotaxime and gentamicin were obtained from Macklin (Shanghai, China); piperacillin and doxycycline were from Aladdin (Shanghai, China); and meropenem was from Solarbio (Beijing, China).

### Growth curve

A bacterial suspension of AB2-1 and EA2-2 at a concentration of 10^6^ colony-forming units (CFU)/mL was mixed in different proportions or subjected to various antibiotic treatments. Following this, 200 µL of the bacterial suspension, with or without antibiotics, was dispensed into each well of a 96-well plate. The plate was then placed in a microplate reader and incubated at 37°C, shaking every 30 min for a total of 20 h. Readings of optical density at a wavelength of 570 nm were taken every half hour, and bacterial growth curves were generated using GraphPad Prism 9.0. Exponential-phase (4–12 h) growth rates were calculated by linear regression of log-transformed OD570 values, and rates from independent biological replicates were compared using an unpaired two-tailed Student’s *t*-test.

### Quantitative determination of biofilms

Single isolated colonies of AB2-1 and EA2-2 were introduced into 5 mL of fresh Luria Bertani (LB) broth and incubated with shaking at 37°C and 180 rpm until they entered the logarithmic growth phase. The two bacterial suspensions were mixed in different proportions. Then, 200 μL of either the mixed or individual bacterial suspension was pipetted into each well of a 96-well plate, with three replicates per group, followed by incubation at 37°C for 48 h. After discarding the unattached (planktonic) cells in the wells, the plates were rinsed three times using phosphate-buffered saline (PBS) at a pH of 7.0 and permitted to dry. Subsequently, the 96-well plates received a 1% solution of crystal violet staining, which was allowed to remain for 15 min for staining purposes. After discarding the staining solution, the plates were washed with distilled water until colorless and then dried again. Finally, 200 μL of absolute ethanol was introduced to completely dissolve the crystal violet, and the resultant solution was moved to a fresh 96-well plate. The biofilm content was assessed by measuring the absorbance at a wavelength of 570 nm with a microplate reader (Thermo Fisher).

### Laser confocal scanning microscopy (CLSM)

The AB2-1 and EA2-2 bacterial suspensions in the logarithmic growth phase were adjusted to 10^6^ CFU/mL and combined in a 1:1 ratio. Each single- and co-culture suspension (1.5 mL) was transferred into a cell culture dish and maintained at 37°C for 24 h. After discarding the culture medium, the samples were slowly washed three times with PSB buffer. Thereafter, 10 μL of the LIVE/DEAD Bacterial Staining Kit with DMAO & PI (86, 88–90) working solution was added to the culture dish, and the staining was carried out for 15 min in the absence of light. Next, the samples were placed under a Laser Confocal Microscope (Leica) for microscopic observation and image acquisition, using 488 and 561 nm laser channels for detection, where viable bacteria appeared green and non-viable cells were visualized in red.

### Scanning electron microscopy (SEM)

A bacterial suspension containing 0.5 McFarland turbidity of AB2-1 and EA2-2 was inoculated at 1% into 5 mL of fresh LB medium. Cell coverslips were adhered to the bottom of six-well plates, and monocultures and 1:1 mixed bacterial suspensions were added, followed by incubation at 37°C for 24 h. After discarding the culture medium, the samples were gently washed three times with PSB buffer. Subsequently, 2.5% glutaraldehyde was added for fixation at 4°C for 4 h. The plates were then washed three times with PBS, followed by dehydration in 50%, 70%, 80%, 90%, and 95% ethanol twice, and anhydrous ethanol three times, with each dehydration step lasting 10 min. Following air drying, gold sputter-coating was applied, and the specimens were analyzed with a cold field emission scanning electron microscope (SU-8010).

### RT-qPCR

AB2-1 and EA2-2 were inoculated in LB broth and cultured overnight. After adjusting the bacterial suspension concentration to 5 × 10^5^ CFU/mL, they were mixed in a 1:1 ratio and transferred to 5 mL of fresh medium at a 1% ratio. Cultivation was carried out at 37°C with agitation at 180 rpm until the cultures reached the logarithmic phase. A 600-μL aliquot was subsequently collected and pelleted by centrifugation at 12,000 rpm for 10 min, after which the supernatant was removed. Total RNA from AB2-1 and EA2-2 was isolated using the RNA Pure Bacteria Kit (Tiangen), followed by DNase I treatment to remove residual DNA contamination. The purified RNA was then reverse-transcribed into cDNA employing either random hexamers or oligo-dT primers. The synthesized cDNA was then mixed with specific primers and SYBR Green fluorescent dye and placed in a real-time PCR instrument for amplification reactions. For qPCR, gDNA extraction was performed with a total reaction volume of 10 μL, and the gDNA template was 5 ng. The cycling program consisted of an initial denaturation at 95°C for 2 min, followed by 40 amplification cycles of 95°C for 5 s and 60°C for 10 s. In the experiment, the biofilm-related genes *pgaB*, *gacS*, *bap*, and *bfm*R from AB2-1, as well as the biofilm-related genes *algI*, *algU*, and *motD* from EA2-2, were selected for transcriptome validation. Each sample was set up with three biological replicates, using the 16S rRNA gene as the reference gene to normalize the relative transcription levels of the studied genes. Relative gene expression levels were calculated using the ΔΔCt method. Primer sequences for RT-qPCR are listed in Table 5.

**TABLE 5:** Primer sequences used for RT-qPCR

Primer	Sequence (5′–3′)
AB-16SrRNA-F	GTTGTGGCTTTAGGTTTATTATACG
AB-16SrRNA-R	AAGTTACTCGACGCAATTCG
pgaB-F	ATTTCCCGAACCGTCATA
pgaB-R	CCAATAGAGGCATCCAAG
bap-F	GGTGTTTCACTTGGTGCGAC
bap-R	GCCTGTATCCGCTACTCCAC
bfmR-F	ACCGATGGTAACCGTGCAAT
bfmR-R	GGGCGAACTTCACGACAAAC
gacS-F	ATGCCATCAAGTTCACGCCAGAC
gacS-R	TGAAGCAAGCACTGACCGATGTC
EA-16SrRNA-F	AATGGGAATTGACAGACGCAG
EA-16SrRNA-R	AACCTAACACCTCACGGCAC
algI-F	TGGATGATGCTTACCGGAAAGGC
algI-R	CCCCAAACTCCGGCATCGAAG
algU-F	TCTCCGAAGACCCGTGAAGAGG
algU-R	AACCACCAGCCTTCTGATATGCC
motD-F	AACTGGGATCTGTCTGCATTGCG
motD-R	TCTGAACGTCCTCCGGCTGTC

### *In vivo galleria mellonella* trials

Logarithmic growth phase cultures of AB2-1 and EA2-2 were resuspended in PBS buffer and adjusted to a concentration of 10^6^ CFU/mL to prepare bacterial suspensions. Final-instar Galleria mellonella larvae were used for infection experiments. Larvae were selected to be creamy white in appearance, highly motile, and capable of a strong righting response, with a body length of approximately 2–3 cm and an average weight of ~300 mg. The bacterial suspensions were mixed in a 1:1 ratio and injected into the left penultimate leg of *Galleria mellonella* larvae. Each strain was tested on 10 larvae, with three biological replicates established. After injection, the larvae were incubated in a dark environment at 37°C. The survival of the larvae in both the single-culture and co-culture groups was recorded every 12 h, and continuous monitoring was conducted for 72 h. Larval death was defined by melanization of the body and the absence of any response to stimulation with blunt forceps. Survival curve analysis was performed using GraphPad 9.0 software.

### Whole genome sequencing and genomic analysis

After extracting the genomic DNA of AB2-1 and EA2-2, the purity, concentration, and integrity were assessed using Nanodrop, Qubit, and 0.35% agarose gel electrophoresis. Subsequently, the SQK-LSK109 ligation kit was employed to construct the library, and after quantifying the library DNA with Qubit, sequencing was performed using an Illumina NovaSeq 6000 platform for short-read sequencing and an Oxford Nanopore platform for long-read sequencing. Raw data filtered by Mecat2 were assembled using Unicycler (https://github.com/rrwick/Unicycler). For the assembled genome, gene prediction was performed using Glimmer3 (91) or Prodigal (92) (with a GC content greater than 70). tRNA was predicted using tRNAscan-SE (Version 1.3.1, http://lowelab.ucsc.edu/tRNAscan-SE/) and rRNA was predicted using rRNAmmer (Version 1.2, http://www.cbs.dtu.dk/services/RNAmmer/). Gene functional annotation was conducted through BLAST and DIAMOND alignments, selecting the optimal alignment results based on identity, e-value, and score. Antibiotic resistance gene annotations were referenced from the CARD database (https://card.mcmaster.ca/), and virulence factor annotations were referenced from the Pathogenic Bacteria Virulence Factors Database (VFDB, http://www.mgc.ac.cn/VFs/main.htm).

### RNA sequencing and transcriptome analysis

RNA sequencing and transcriptome analysis were conducted using total RNA extracted from AB2-1 and EA2-2 via the Trizol method. RNA quality and integrity were verified with a NanoDrop One spectrophotometer (Thermo) and an Agilent 4200 TapeStation. Ribosomal RNA was depleted from high-quality samples using the Ribo-Zero rRNA Removal Kit (Epicenter). Strand-specific cDNA libraries were then generated with the NEBNext Ultra II Directional RNA Library Prep Kit (Illumina) and sequenced on the Illumina NovaSeq platform with paired-end 150 bp reads. The raw images obtained from sequencing were transformed into raw sequencing reads through base calling analysis. The quality of the sequencing data were evaluated, and low-quality data were filtered using the fastp software (93). Transcript-level quantifications were summarized to gene-level counts via the tximport package in R (94). Differential gene expression analysis was executed using edgeR (95, 96) software. Genes with an absolute log2 fold change greater than 1 and an adjusted *P*-value below 0.05 were considered significantly differentially expressed. Further functional annotation was performed through comprehensive databases such as Gene Ontology (GO) and the Kyoto Encyclopedia of Genes and Genomes (KEGG), with GO and KEGG functional enrichment analyses conducted using the clusterProfiler software.

## Supplementary Material

Reviewer comments

## Data Availability

The genomic data sets generated in this work have been deposited in the National Center for Biotechnology Information (NCBI) under BioProject accession numbers PRJNA1311550 and PRJNA1311553.

## SUPPLEMENTAL MATERIAL

Data S1 spectrum.03349-25-s0001.docx Detailed clinical characteristics of 15 patients with *A. baumannii* and *Elizabethkingia* co-infection.

Data S2 spectrum.03349-25-s0002.xlsx Antibiotic administration during hospitalization in 15 patients.

Data S3 spectrum.03349-25-s0003.xlsx Resistance factors of *A. baumannii* AB2-1 and *E. anophelis* EA2-2.

Data S4 spectrum.03349-25-s0004.xlsx Virulence factors of *A. baumannii* AB2-1 and *E. anophelis* EA2-2.

Fig. S1 spectrum.03349-25-s0005.docx Colony growth of AB2-1 and EA2-2 under mono- and co-culture conditions.

OPEN PEER REVIEW reviewer-comments.pdf An accounting of the reviewer comments and feedback.

ASM does not own the copyrights to Supplemental Material that may be linked to, or accessed through, an article. The authors have granted ASM a non-exclusive, world-wide license to publish the Supplemental Material files. Please contact the corresponding author directly for reuse.
