# Supplementary material for: Biofilm-mediated antibiotic cross-protection: Acinetobacter baumannii-driven enhancement of Elizabethkingia anopheles
Source: Microbiol Spectr. 2026 Jun 16;14(7):e03349-25. doi: 10.1128/spectrum.03349-25 (PMC13340172; doi:10.1128/spectrum.03349-25)
Supplement: Data S1 — Detailed clinical characteristics of 15 patients with A. baumannii and Elizabethkingia co-infection. [file spectrum.03349-25-s0001.docx]

**Supplementary Data 1.** Detailed clinical characteristics of 15 patients with A. baumannii and Elizabethkingia co-infection.

| Patient ID | Age | Sex | Comorbidities | Main diagnosis | *^2^Invasive procedures per patient, n | Length of hospital stay | Outcome |
| --- | --- | --- | --- | --- | --- | --- | --- |
| P01 | 84 | *^1^M | Hypertension,  Ischemic stroke,  Intracerebral hemorrhage. | Pulmonary infection,  Respiratory failure,  Cerebrovascular disease,  Gastrointestinal bleeding. | 5 | 8 | *^3^DAMA |
| P02 | 33 | F | None | Acute pulmonary embolism (PE),  Cardiorespiratory arrest,  Intracranial complications,  Pulmonary complications,  Femoral arteriovenous fistula,  Multiple organ dysfunction syndrome (MODS),  Disseminated intravascular coagulation (DIC). | 9 | 11 | Death |
| P03 | 71 | M | Hypertension,  Ischemic stroke. | Pulmonary infection,  Respiratory failure,  Lower extremity deep vein thrombosis (DVT). | 4 | 19 | DAMA |
| *^4^P04 | 58 | M | Diabetes mellitus,  Status post aortic valve bioprosthesis replacement. | Severe pneumonia,  MODS,  Septic shock. | 5 | 25 | Death |
| P05 | 52 | M | Hypertension,  Intracerebral hemorrhage. | Ruptured intracranial aneurysm with Subarachnoid hemorrhage,  Pulmonary infection,  Respiratory failure. | 5 | 27 | Transferred to rehabilitation |
| P06 | 75 | F | Hypertension,  Old pulmonary tuberculosis. | Pulmonary infection,  Sepsis with septic shock,  Idiopathic pulmonary fibrosis (IPF),  Acute respiratory distress syndrome (ARDS). | 6 | 30 | DAMA |
| P07 | 64 | M | Hypertension | Intracerebral hemorrhage,  Severe pneumonia,  Acute liver failure,  ARDS. | 5 | 34 | Discharged |
| P08 | 81 | M | Hypertension | Pulmonary infection,  ARDS,  Cardiovascular failure. | 4 | 34 | Transferred to rehabilitation |
| P09 | 78 | F | Diabetes mellitus,  Ischemic stroke,  Atrial fibrillation . | Femoral neck fracture,  Sepsis with septic shock,  Pulmonary infection,  Respiratory failure. | 5 | 39 | DAMA |
| P10 | 53 | M | Hypertension,  Diabetes mellitus. | Severe acute pancreatitis,  Pulmonary infection,  ARDS,  Hyperlipidemia. | 5 | 49 | Discharged |
| P11 | 70 | M | Hypertension,  Diabetes mellitus,  Ischemic stroke. | Multiple trauma with multiple fractures,  ARDS,  Uremic shock. | 6 | 41 | DAMA |
| P12 | 87 | F | Status post coronary stent implantation. | Acute myocardial infarction (AMI),  Cardiac arrest with return of spontaneous circulation (ROSC),  Respiratory failure,  Sepsis. | 6 | 56 | DAMA |
| P13 | 48 | M | Hypertension,  Ischemic stroke. | Acute pancreatitis,  ROSC,  Hypoxic-ischemic encephalopathy (HIE),  Multiple organ failure (MOF). | 4 | 76 | DAMA |
| P14 | 80 | M | Hypertension | Pulmonary infection,  Atelectasis,  Pleural effusion,  MODS. | 6 | 80 | Death |
| P15 | 58 | M | Diabetes mellitus. | Cerebral contusion,  Traumatic small bowel perforation,  ROSC,  Pulmonary infection,  Respiratory failure,  HIE,  Intra-abdominal abscess,  Septic shock, DVT. | 7 | 89 | Transferred to rehabilitation |

*1: M = male; F = female; ICU = intensive care unit;

*2: Invasive procedures include vascular catheterization; indwelling urinary catheterization; nasogastric tube insertion; upper limb arterial exploration; femoral artery stent placement; endotracheal intubation/tracheostomy; fiberoptic bronchoscopy; extracorporeal membrane oxygenation (ECMO) support; thoracentesis and drainage; intracranial surgery; and thoracic surgery; intramedullary nailing for fracture; small bowel surgery.

*3: DAMA = discharged against medical advice.
*4: The *A. baumannii* and *E. anophelis* strains isolated from this patient were used for subsequent in vitro experiments in this study.
