# Supplementary material for: Biofilm-mediated antibiotic cross-protection: Acinetobacter baumannii-driven enhancement of Elizabethkingia anopheles
Source: Microbiol Spectr. 2026 Jun 16;14(7):e03349-25. doi: 10.1128/spectrum.03349-25 (PMC13340172; doi:10.1128/spectrum.03349-25)
Supplement: Fig. S1 — Colony growth of AB2-1 and EA2-2 under mono- and co-culture conditions. [file spectrum.03349-25-s0005.docx]

**Supplementary Figure 1. Colony growth of AB2-1 and EA2-2 under mono- and co-culture conditions.**

**
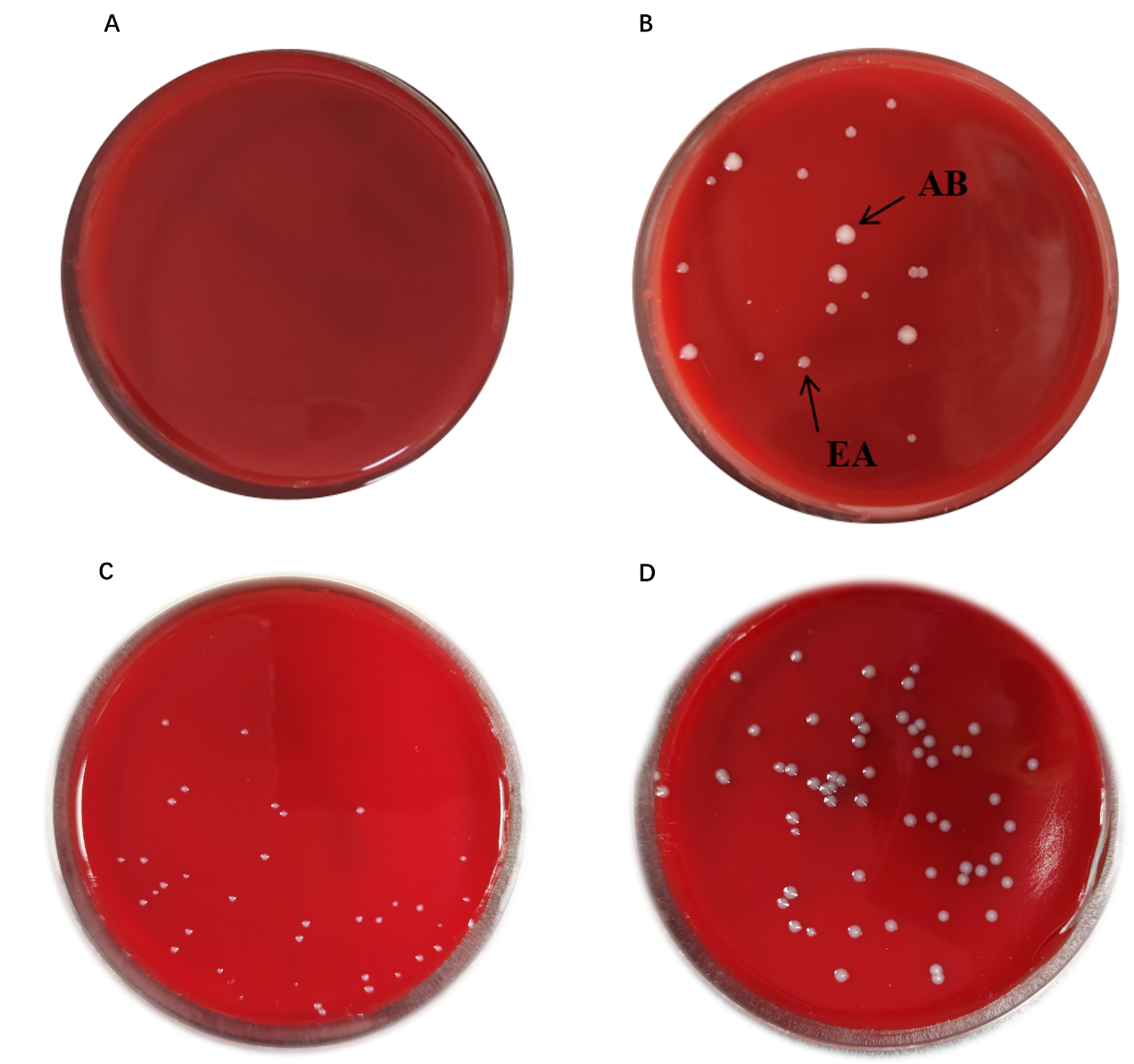
**

(A) EA2-1 mono-culture treated with 64 μg/mL doxycycline, with no colony growth observed. (B) AB2-1 and EA2-2 co-culture treated with 64 μg/mL doxycycline, with colonies of both strains observed. (C) AB2-1 and EA2-2 co-culture treated with 128 μg/mL gentamicin, with colonies of only a single strain grown. (D) AB2-1 and EA2-2 co-culture treated with 256 μg/mL meropenem, with colonies of only a single strain grown.
