# Supplementary material for: Biofilm-mediated antibiotic cross-protection: Acinetobacter baumannii-driven enhancement of Elizabethkingia anopheles
Source: Microbiol Spectr. 2026 Jun 16;14(7):e03349-25. doi: 10.1128/spectrum.03349-25 (PMC13340172; doi:10.1128/spectrum.03349-25)
Supplement: Reviewer comments [file reviewer-comments.pdf]

Biofilm-mediated Antibiotic Cross-protection: *Acinetobacter baumannii*-driven Enhancement of *Elizabethkingia anophelis*

Dejin Pan, Ren Liu, Jiehui Qiu, Ziyi WU, Nan SU, jianglong shi, and Na Cheng

Corresponding Author(s): Na Cheng, Department of Infectious Disease, the First Affiliated Hospital of Nanchang University

Review Timeline:

Submission Date:	October 28, 2025
Editorial Decision:	December 23, 2025
Revision Received:	January 26, 2026
Accepted:	February 11, 2026

Editor: Valerie Carabetta

Reviewer(s): Disclosure of reviewer identity is with reference to reviewer comments included in decision letter(s). The following individuals involved in review of your submission have agreed to reveal their identity: Ryath Shaya Al-Hemedawi (Reviewer #1); S Adeeb Mujtaba Ali (Reviewer #2)

Transaction Report:

(Note: With the exception of the correction of typographical or spelling errors that could be a source of ambiguity, letters and reports are not edited. The original formatting of letters and referee reports may not be reflected in this compilation.)

DOI: https://doi.org/10.1128/spectrum.03349-25

Re: Spectrum03349-25 (**Biofilm-mediated Antibiotic Cross-protection: *Acinetobacter baumannii*-driven Enhancement of *Elizabethkingia anophelis***)

Dear Dr. Na Cheng:

Thank you for the privilege of reviewing your work. Below you will find my comments, instructions from the Spectrum editorial office, and the reviewer comments.

Two experts in the field reviewed your work, however, I did not feel the comments of one reviewer were valid or constructive. Since I have appropriate expertise, I served as the second reviewer for your manuscript. My comments are below, and reviewer 1's comments are attached.

For the future, include line numbers to facilitate review.

Please be sure that all species names are italicized (including the references) throughout the manuscript. In addition, the full genus-species name is only needed at the first appearance. Please ensure this convention is proper throughout. Also, before each reference citation, there should be a space.

Introduction:
Paragraph 1, "....This opportunistic pathogen is capable...and easily colonizing patients in healthcare settings." Is that what you meant? The sentence as written sounds odd. Last sentence of the paragraph, change to, "However, how the mechanisms..."

Paragraph 3: What do you mean by "most sever prognosis?"

Results
Clinical Data: 4th line down, it should be "hospitalization."

In Table 2, why were those drugs selected for analysis? Is that considered standard by the hospital? If so, can this be mentioned either in the results or methods section to provide a rationale?

In the section where the patient information is described, can you mention/reference which strain in supplemental data 2 is that particular patient? When describing the drugs used, you do not need to include "sodium." So just list piperacillin-tazobactam, and ampicillin.

MBLs do not confer resistance to aminoglycosides. Please correct this statement (paragraph below table 3, 9 lines down). Also, from Table 2, your Ab2-1 stain qualifies as MDR, as it is resistant to at least 1 drug in 3 or more drug classes (beta-lactams, beta lactam/beta-lactamase inhibitor combos, fluoroquinolones). You should discuss the tetracycline resistance mechanisms in each strain, as you will use that drug later for the coculture experiments.

When mentioning genes, they should be all lowercase, except for the last letter, and in italics. Please correct throughout.
For the virulence factors, there also is a somewhat large difference in adherence genes between AB2-1 and EA2-2. This could be elaborated on.

Below figure 1: Delete the "sodium" following drug names.

Cross protection studies:
On the page with Table 4, top paragraph. If these are shaking cultures in a plate reader, is that in the context of biofilm?

What does the AUC refer to? I think it would be better to calculate and report growth rates (doubling times). You could do statistical tests as well. I'm not sure the growth rates are significantly different in doxycycline condition. The ceftazidime results probably are likely due to the increased destruction of the drugs by all the beta-lactamases secreted by the two strains.

In the next paragraph, it is mentioned that "no distinct colonies were observed on plates treated with gentamicin and meropenem. That is not true. There are colonies on each of those plates, but they are uniform, representing only 1 species (as predicted by the data in Table 4). These do not demonstrate cross-protection, and it should be mentioned that EA2-2 does not protect AB!

Right above table 4, that paragraph mentions "significantly higher" (figure 2B), but not statistical analysis was performed. Be careful with language. This data also should be analyzed by doubling times.

Consider renumbering figure 3 and 4 so that they are talked about in numerical order in the text.

In the second paragraph below figure 2, The beta-lactamases do not confer resistance to tetracylcines. You should discuss the data related to efflux pumps here, especially TetB (a known tetracycline efflux pump). Where any of those differentially expressed? Add this data to figure 3.

Do you find the decrease in biofilm promoting genes in EA2-2 surprising? What are your ideas as to why this is the case? Do you think it lets AB do all the work, almost like an evolutionary "cheater?"

Consider moving Figure 5B and C to become a new figure 7, so that the data can be talked about in order in the text.

Discussion:
Do coinfections of AB-2-1 and EA2-2 "frequently" occur? Maybe soften the language to "can occur."

It is mentioned that after co-culturing, MexA, BepF/E and OprM and OatA were overexpressed. That data should be included.

Your last section about future suggestions is good but could be expanded upon. I was thinking quorum sensing would be interesting to follow up on. Could you add a little bit of discussion on this, as QS likely determines how the bacteria interact.

Materials and Methods
For the clinical data, was there any IRB or Ethics committee approval? Please add details.

Define MH as "Mueller Hinton"

Add info pertaining to where antibiotics were purchased from.
Can you use the most up-to-date CLSI version?

Growth curve section: Define CFU at first appearance here, then abbreviate. "...bacterial suspension or the antibiotic mixture..." Do you mean bacteria with or without antibiotics, or do you not mean either, and mean bacteria and antibiotics? Please clarify.

By "bacterial liquid" do you mean "unattached cells?"

RT-qPCR: what probes and fluorescent dyes were used? Add more details. Which methods did you use for calculations (relative standard curve, delta delta Ct)? Also, that is Table 5, not supplemental table 5.

Whole genome sequencing section: Change to: "After extracting the genomic DNA of..." What instrument was used for sequencing?

Please return the manuscript within 60 days; if you cannot complete the modification within this time period, please contact me. If you do not wish to modify the manuscript and prefer to submit it to another journal, notify me immediately so that the manuscript may be formally withdrawn from consideration by Spectrum.

Revision Guidelines
To submit your modified manuscript, log into the submission site at https://spectrum.msubmit.net/cgi-bin/main.plex. Go to Author Tasks and click the appropriate manuscript title to begin. The information you entered when you first submitted the paper will be displayed; update this as necessary. Note the following requirements:

• Upload point-by-point responses to the issues raised by the reviewers in a file named "Response to Reviewers," NOT in your cover letter.
• Upload a compare copy of the manuscript (without figures) as a "Marked-Up Manuscript" file.
• Upload a clean .DOC/.DOCX version of the revised manuscript and remove the previous version.
• Each figure must be uploaded as a separate, editable, high-resolution file (TIFF or EPS preferred), and any multipanel figures must be assembled into one file.
• Any supplemental material intended for posting by ASM should be uploaded with their legends separate from the main manuscript. You can combine all supplemental material into one file (preferred) or split it into a maximum of 10 files with all associated legends included.

For complete guidelines on revision requirements, see our Submission and Review Process webpage. Submission of a paper that does not conform to guidelines may delay acceptance of your manuscript.

Data availability: ASM policy requires that data be available to the public upon online posting of the article, so please verify all links to sequence records, if present, and make sure that each number retrieves the full record of the data. If a new accession number is not linked or a link is broken, provide Spectrum production staff with the correct URL for the record. If the accession numbers for new data are not publicly accessible before the expected online posting of the article, publication may be delayed;

please contact production staff (Spectrum@asmusa.org) immediately with the expected release date.

Publication Fees: For information on publication fees and which article types are subject to charges, visit our website. If your manuscript is accepted for publication and any fees apply, you will be contacted separately about payment during the production process; please follow the instructions in that e-mail. Arrangements for payment must be made before your article is published.

ASM Membership: Corresponding authors may join or renew ASM membership to obtain discounts on publication fees. Need to upgrade your membership level? Please contact Customer Service at Service@asmusa.org.

The ASM Journals program strives for constant improvement in our submission and publication process. Please tell us how we can improve your experience by taking this quick Author Survey.

Thank you for submitting your paper to Spectrum.

Sincerely,
Valerie Carabetta
Editor
Microbiology Spectrum

The feedback on the paper titled:

Biofilm-mediated Antibiotic Cross-protection: Acinetobacter baumannii-driven Enhancement of Elizabethkingia anophelis.

1. Is the study limited to elderly patients, or does it involve participants of all ages?
2. Based on the patient's history, do they reside in a rural or urban area? You noted that mosquitoes transmit Elizabethkingia.
3. I believe the study should place greater emphasis on the mechanisms underlying the interactions between microorganisms, as mixed infections are known to be challenging to treat.
4. In the text under Table 2, line 10, a repetition was noted (from).
5. For bacterial fitness, did you compare the growth of your strains (wild-type and mutated)?
6. In Figure 5A, a control medium was not used; similarly, for Figures B and C, there was no wild-type bacteria or control medium included to compare with the mutated strains.
7. Regarding Figure 6, I suggest that using a 96-well plate is more precise and reliable than a tube biofilm assay. Additionally, you should repeat the experiment comparing the mutated strain with the wild type and include media as a control.
8. In Figure 7, there is no comparison between your strain and the mutated strain, nor is there a control included. Additionally, please consider incorporating images to illustrate the larvae, both live and dead, along with their symptoms observed during the experiments.
9. The following link provides a more accurate method for measuring bacterial growth: https://www.labtools.us/bacterial-cell-number-od600/

Bes of luck

Dr. Ryath Al-Hemedawi

Response to the Reviewers' Comments

Title: Biofilm-mediated Antibiotic Cross-protection: *Acinetobacter baumannii*-driven Enhancement of *Elizabethkingia anophelis*

Dear Editors and Reviewers,

We thank the Editor and the reviewers for their careful evaluation of our manuscript and for the constructive comments provided. These comments were helpful in improving the manuscript. We have reviewed each suggestion and revised the manuscript accordingly. We now submit the revised version for your consideration. Below, we provide point-by-point responses to each comment.

Comment 1: For the future, include line numbers to facilitate review.

Response 1:

As suggested, line numbers have been added to the revised manuscript.

Comment 2: Please be sure that all species names are italicized (including the references) throughout the manuscript. In addition, the full genus-species name is only needed at the first appearance. Please ensure this convention is proper throughout. Also, before each reference citation, there should be a space.

Response 2:

All species names have been carefully checked and revised throughout the manuscript, including the reference list, to ensure that they are consistently italicized. The full genus–species names *Acinetobacter baumannii* and *Elizabethkingia spp.* are retained at their first appearance in the manuscript (lines 15 and 16, respectively). Because the genus *Elizabethkingia spp.* includes multiple species, the full species name *Elizabethkingia anophelis* is retained at its first occurrence (line 22). Thereafter, *A. baumannii* and *E. anophelis* are used as abbreviations at their first abbreviated appearances (lines 23 and 31, respectively). Furthermore, the study strains are introduced as *A. baumannii* strain AB2-1 and *E. anophelis* strain EA2-2 at their first mention (lines 136 and 137, respectively), and are subsequently referred to as AB2-1 and EA2-2 for clarity and consistency.

Finally, a space has been added before each reference citation throughout the manuscript, as requested.

Introduction:

Comment 3: Paragraph 1, "....This opportunistic pathogen is capable...and easily colonizing patients in healthcare settings." Is that what you meant? The sentence as written sounds odd. Last sentence of the paragraph, change to, "However, how the mechanisms..."

Response 3:

We agree that the original sentence was awkwardly phrased and have revised it to improve clarity and accuracy. Now we have revised the sentence to improve clarity

and accuracy as follows: "This opportunistic pathogen is capable of forming biofilms, surviving for extended periods in moist environments, and readily colonizing healthcare settings."
In addition, as recommended, we have revised the last sentence of the paragraph to: "However, how the antibiotic resistance mechanisms interact with and influence virulence changes following co-infection remains poorly understood."

Comment 4: Paragraph 3: What do you mean by "most severe prognosis?"
Response 4:
We recognize that the term "most severe prognosis" was insufficiently defined in the original manuscript and may cause ambiguity. All patients included in this study were critically ill ICU patients who required invasive interventions; therefore, the selected case was clinically consistent with the overall study population and did not represent an atypical scenario. In this context, we acknowledge that the use of the term "most severe prognosis" was not sufficiently precise, and it has been removed from the revised manuscript.
In the revised version, we have clarified the rationale for selecting this patient-derived strain pair for subsequent experiments in the Materials and Methods section under "Bacterial Strains and Growth Conditions."
Both *Acinetobacter baumannii* and *Elizabethkingia anophelis* were isolated from the same sputum specimen, which better reflects their true coexistence within the host and minimizes the possibility of sequential secondary infection or contamination. Moreover, the antimicrobial resistance profiles of these two isolates were consistent with those observed in the majority of coinfected cases included in this study (Table 2), supporting their representativeness. Therefore, the selection of this strain pair for subsequent in vitro coculture experiments was based on comparable clinical context, clear evidence of coinfection, and representative resistance phenotypes.

Results
Comment 5: Clinical Data: 4th line down, it should be "hospitalization."
Response 5:
The typo has been corrected, and "hospitalizatio" has been revised to "hospitalization".

Comment 6: In Table 2, why were those drugs selected for analysis? Is that considered standard by the hospital? If so, can this be mentioned either in the results or methods section to provide a rationale?
Response 6:
The antibiotics listed in Table 2 represent the standardized antimicrobial susceptibility testing panel routinely used in our hospital for Gram-negative clinical isolates. This panel includes the major antibiotic classes commonly used for the treatment of nosocomial infections. We have now clarified this rationale in the Materials and Methods section under "Clinical Data Collection" in the revised manuscript.

Comment 7: In the section where the patient information is described, can you mention/reference which strain in supplemental data 2 is that particular patient? When describing the drugs used, you do not need to include "sodium." So just list piperacillin-tazobactam, and ampicillin.
Response 7:
In the Results section, we have clarified that the experimental strains were isolated from patient 04 (P04) listed in Supplemental Data 1-2. In addition, Supplemental Data 1-2 has been updated to indicate that the *A. baumannii* and *E. anophelis* strains isolated from this patient were used for subsequent in vitro experiments in this study.
Regarding drug nomenclature, the term "sodium" has been removed, and the drugs are now listed as piperacillin-tazobactam and ampicillin, as suggested.

Comment 8: MBLs do not confer resistance to aminoglycosides. Please correct this statement (paragraph below table 3, 9 lines down). Also, from Table 2, your Ab2-1 stain qualifies as MDR, as it is resistant to at least 1 drug in 3 or more drug classes (beta-lactams, beta lactam/beta-lactamase inhibitor combos, fluoroquinolones). You should discuss the tetracycline resistance mechanisms in each strain, as you will use that drug later for the coculture experiments.
Response 8:
We concur with the reviewer that metallo-β-lactamases do not confer resistance to aminoglycosides. As a result, we have corrected the sentence by removing the reference to aminoglycoside resistance.
Additionally, AB2-1 qualifies as MDR. To avoid ambiguity, the phrase "potentially leading to multidrug resistance" has been revised to "and could lead to multidrug resistance (MDR)" in the manuscript.
Furthermore, because tetracycline-class antibiotics are used in subsequent coculture experiments, we have added a description of the tetracycline resistance mechanisms in both strains (lines 313-318). "Genomic analysis under monoculture conditions revealed that AB2-1 harbors the *tetB* and *tetR* genes (Figure 1A), which are closely associated with bacterial resistance to tetracycline-class antibiotics (51, 52). By comparison, no tetracycline-related resistance determinants were identified in the genome of EA2-2 (Figure 1B and Supplementary Data 3), indicating a genetic basis for differential susceptibility to tetracyclines between the two strains." Relevant references have been cited in the revised manuscript.

Comment 9: When mentioning genes, they should be all lowercase, except for the last letter, and in italics. Please correct throughout.
Response 9: All gene names throughout the manuscript have been corrected to follow standard nomenclature, with gene symbols italicized and formatted in lowercase, except for the final letter.

Comment 10: For the virulence factors, there also is a somewhat large difference in adherence genes between AB2-1 and EA2-2. This could be elaborated on.
Response 10:

We have expanded the description of the differences in adherence-related virulence factors between AB2-1 and EA2-2 in the revised manuscript (lines 199-210). Specifically, the revised text states: "Beyond such regulatory mechanisms, adhesion factors themselves play a crucial role in bacterial colonization and persistent infection. The adhesion factors of both strains involve various structures such as flagella, pili, and membrane proteins, which aid in their colonization on host surfaces and biofilm formation, constituting a key step in initiating infection (35-37). In comparison, AB2-1 possesses a greater variety and number of adhesion factors, particularly a significant increase in genes related to type IV pili, as well as multiple pilus systems associated with surface attachment and biofilm formation, such as AF/R1 pili and MR/P fimbriae (38, 39). In contrast, EA2-2 has relatively fewer adhesion factors, primarily consisting of non-specific adhesion proteins like EF-Tu and Hsp60 (40, 41), and shows a relative deficiency in pilus systems. This difference suggests that AB2-1 may possess stronger colonization capacity and competitive advantage." Relevant references have been cited in the revised manuscript.

Comment 11: Below figure 1: Delete the "sodium" following drug names.
Response 11:
Corrected as suggested, "sodium" has been removed from the drug names below Figure 1.

Cross protection studies
Comment 12: On the page with Table 4, top paragraph. If these are shaking cultures in a plate reader, is that in the context of biofilm?
Response 12:
The growth curves shown in Figure 2A were obtained from 96-well plates with intermittent shaking in a plate reader, representing the growth of planktonic cells rather than mature biofilms. The manuscript has been revised accordingly to clarify the experimental conditions and avoid potential confusion. The text now states (lines 249-250): "we performed coculture growth assays under planktonic conditions in the presence of 64 μg/mL doxycycline."

Comment 13: What does the AUC refer to? I think it would be better to calculate and report growth rates (doubling times). You could do statistical tests as well. I'm not sure the growth rates are significantly different in doxycycline condition. The ceftazidime results probably are likely due to the increased destruction of the drugs by all the beta-lactamases secreted by the two strains.
Response 13:
The area under the curve (AUC) represents the integral of OD570 values over time and was used as a composite measure reflecting overall bacterial growth during the incubation period. For greater rigor, the AUC-related statements have been removed. Exponential-phase growth rates were calculated by linear regression of log-transformed OD values between 4 and 12 h (lines 252-257). "Under doxycycline exposure, the growth rate of AB2-1 in coculture with EA2-2 ($0.178 \pm 0.004\ h^{-1}$)

was higher than that observed in monoculture (0.136 ± 0.005 h^{-1}), and this difference was statistically significant (unpaired t-test, $p < 0.001$)". Details of the statistical methods have also been added to the Materials and Methods section under "Growth curves" (lines 591-593).
Regarding the observation that "the maximum biomass (OD570) of the co-culture exposed to cefotaxime remained higher than that of the monoculture", we agree with the reviewer's assessment. Both AB2-1 and EA2-2 are resistant to β-lactam antibiotics, and the increased maximum biomass in the co-culture is likely attributable, at least in part, to the degradation of the antibiotic facilitated by β-lactamases produced by both strains. The text now states (lines 260-263): "the maximum biomass (OD570) of the co-culture exposed to cefotaxime remained higher than that of the monoculture, which is likely attributable, at least in part, to the degradation of the antibiotic facilitated by β-lactamases produced by both strains."

Comment 14: In the next paragraph, it is mentioned that "no distinct colonies were observed on plates treated with gentamicin and meropenem. That is not true. There are colonies on each of those plates, but they are uniform, representing only 1 species (as predicted by the data in Table 4). These do not demonstrate cross-protection, and it should be mentioned that EA2-2 does not protect AB!
Response 14:
We apologize for the unclear wording. Our intention was to indicate that no distinct colony morphologies corresponding to two different species were observed on plates treated with gentamicin or meropenem. In fact, colonies were present on these plates, but they were morphologically uniform and represented a single species, consistent with the susceptibility profiles shown in Table 4. We have revised the text accordingly.
Under these conditions, EA2-2 did not protect AB2-1. We have revised the text to specify that the cross-protection effect was observed exclusively under doxycycline treatment, a scenario characterized by a marked difference in antibiotic susceptibility between the two strains.We have revised the text to read (lines 272-279): "Taken together, these results indicate that cross-protection in coculture occurs only under antibiotic conditions in which there is a pronounced difference in susceptibility between the two strains. More specifically, under doxycycline treatment, AB2-1 protected EA2-2 from antibiotic exposure, whereas no cross-protection was observed under gentamicin or meropenem treatment. These findings suggest that, in coculture, when a marked difference in antibiotic resistance exists, AB2-1 may mitigate the inhibitory effects of antibiotics on EA2-2 through an interspecies interaction-dependent mechanism."

Comment 15: Right above table 4, that paragraph mentions "significantly higher" (figure 2B), but not statistical analysis was performed. Be careful with language. This data also should be analyzed by doubling times.
Response 15:
We thank the reviewer for this helpful comment, and we have revised the text to avoid

implying statistical significance where none was performed. The sentence now reads : "The results showed that co-cultures reached a higher maximum biomass (OD value) in the stationary phase compared to monocultures (Figure 2B)."

Because the 1:1 inoculation ratio represents the most balanced co-culture condition and shows the most pronounced and reproducible enhancement in exponential-phase growth, we focused on calculating the growth rate under this condition. We have revised this section in the manuscript to read: "In growth kinetic experiments conducted without drug pressure, logarithmic-phase cultures of the two strains were adjusted to a McFarland turbidity of 0.5, mixed at different ratios, and used to plot 20-hour growth curves. Specifically, when co-cultured at a 1:1 inoculation ratio, the exponential-phase growth rate ($0.178 \pm 0.004\ h^{-1}$) was significantly higher than that of AB2-1 in monoculture ($0.136 \pm 0.005\ h^{-1}$) (unpaired t-test, $p < 0.001$).

Comment 16: Consider renumbering figure 3 and 4 so that they are talked about in numerical order in the text.

Response 16:

Figures 3 and 4 have been renumbered so that they now appear in numerical order as cited in the text.

Comment 17: In the second paragraph below figure 2, The beta-lactamases do not confer resistance to tetracylcines. You should discuss the data related to efflux pumps here, especially *TetB* (a known tetracycline efflux pump). Where any of those differentially expressed? Add this data to figure 3.

Response 17:

We fully agree that β-lactamases do not confer resistance to tetracyclines. In line with this clarification, the relevant paragraph below Figure 2 has been revised (lines 313-334) to remove this implication and to focus on efflux pumps. In particular, based on genomic analysis (Supplementary Data 3), AB2-1 harbors the tetracycline resistance－associated genes *tetB* and *tetR*. We have therefore added the transcriptomic expression levels of *tetB*, *tetR*, as well as representative efflux pump genes (*adeB*, *adeJ*, and *abeM*), to the revised Figure 4.

In contrast, no tetracycline resistance determinants were identified in the genome of EA2-2. Among its efflux-related genes, *qacG/qacJ* are primarily associated with disinfectant resistance; therefore, the expression level of the efflux pump gene *adeF* has been included in Figure 4 for comparison.

Comment 18: Do you find the decrease in biofilm promoting genes in EA2-2 surprising? What are your ideas as to why this is the case? Do you think it lets AB do all the work, almost like an evolutionary "cheater?"

Response 18:

This is indeed a noteworthy observation. In this study, several biofilm-promoting genes in EA2-2 were downregulated under co-culture conditions, and this transcriptional pattern may reflect an adaptive response to a mixed-species biofilm environment. AB2-1 possesses a stronger biofilm-forming capacity and, when

co-cultured with EA2-2, produces increased amounts of extracellular matrix components (as indicated by enhanced expression of PNAG-associated enzymes), which may dominate the structural framework of the co-culture biofilm. In this context, EA2-2 may benefit from the established biofilm architecture and the associated protective microenvironment without maintaining a high level of investment in its own biofilm-related functions, while still sustaining survival. Therefore, this interaction is conceptually more consistent with a division-of-labor strategy rather than active exploitation. The current data do not support the conclusion that EA2-2 deliberately "cheats" AB2-1. Instead, the observed transcriptional changes are more consistent with a passive adaptation of EA2-2 to a shared biofilm environment primarily formed by AB2-1. Whether this interaction confers long-term evolutionary advantages or represents a transient physiological adaptation remains to be determined.

Comment 19: Consider moving Figure 5B and C to become a new figure 7, so that the data can be talked about in order in the text.
Response 19:
We have moved Figure 5B and 5C to a new Figure 7, as recommended. The manuscript text has been updated accordingly to discuss the figures in sequential order.

Discussion:
Comment 20: Do coinfections of AB-2-1 and EA2-2 "frequently" occur? Maybe soften the language to "can occur".
Response 20:
We agree with the reviewer and have softened the language by replacing "frequently occur" with "can occur" to more accurately reflect our data.

Comment 21: It is mentioned that after co-culturing, *MexA*, *BepF/E* and *OprM* and *OatA* were overexpressed. That data should be included.
Response 21:
We have now included the quantitative expression data (log2 fold change values) for the aforementioned genes in the revised manuscript text (lines 482-488).

Comment 22: Your last section about future suggestions is good but could be expanded upon. I was thinking quorum sensing would be interesting to follow up on. Could you add a little bit of discussion on this, as QS likely determines how the bacteria interact.
Response 22:
Thanks for this helpful suggestion. We have expanded the future directions section to include additional discussion on the potential role of quorum sensing in mediating interspecies interactions. (lines 498 – 515)

Materials and Methods

Comment 23: For the clinical data, was there any IRB or Ethics committee approval? Please add details.
Response 23:
Ethical approval information has been added to the manuscript (lines 551-553). The study was reviewed and approved by the Institutional Review Board (Approval No. IIT2024667).

Comment 24: Define MH as "Mueller Hinton"
Response 24:
The abbreviation MH has been defined as Mueller Hinton at its first occurrence in the Materials and Methods section, with agar and broth forms specified where appropriate (lines 565 and 569).

Comment 25: Add info pertaining to where antibiotics were purchased from. Can you use the most up-to-date CLSI version?
Response 25:
We have added information on the sources of the antibiotics used in this study, including the supplier names and locations, in the revised manuscript under the Materials and Methods section ("Antimicrobial susceptibility testing and cross-protection experiments") (lines 580-583).
In addition, the antimicrobial susceptibility testing was performed in November 2024, and MIC values were interpreted according to the CLSI M100, 34th Edition (2024), which was the most up-to-date version available at the time of the experiments. We note that the subsequent CLSI update does not materially alter the interpretation of susceptibility for the antibiotics tested in this study.

Comment 26: Growth curve section: Define CFU at first appearance here, then abbreviate. "...bacterial suspension or the antibiotic mixture..." Do you mean bacteria with or without antibiotics, or do you not mean either, and mean bacteria and antibiotics? Please clarify.
Response 26:
CFU has been defined at its first occurrence in the Materials and Methods section (lines 585-586). Moreover, the description of the growth curve assay has been clarified to specify that bacterial suspensions were measured in the presence or absence of antibiotics (lines 587-588).

Comment 27: By "bacterial liquid" do you mean "unattached cells?"
Response 27:
The term "bacterial liquid" has been replaced with "unattached (planktonic) cells" to clarify that only non-adherent cells were removed prior to biofilm staining (lines 601-602).

Comment 28: RT-qPCR: what probes and fluorescent dyes were used? Add more details. Which methods did you use for calculations (relative standard curve, delta

delta Ct)? Also, that is Table 5, not supplemental table 5.

Response 28:

We have clarified that RT-qPCR was performed using the SYBR Green fluorescent dye method, and relative gene expression levels were calculated using the ΔΔCt method with an internal reference gene. This information has been added to the Materials and Methods section under "RT-qPCR". And table 5 now lists the primer sequences (lines 645 and 654-655).

Comment 29: Whole genome sequencing section: Change to: "After extracting the genomic DNA of..." What instrument was used for sequencing?

Response 29:

The term "whole genome DNA" has been replaced with the standard term "genomic DNA" in this section. And the sequencing instruments have now been specified in the Materials and Methods section. Short-read sequencing was performed using the Illumina NovaSeq 6000 platform, and long-read sequencing was performed using an Oxford Nanopore platform (lines 678-680).

Responses to Reviewer 1

Comment 1: Is the study limited to elderly patients, or does it involve participants of all ages?

Response 1:

We apologize for any potential lack of clarity in the original description. This study was not intentionally limited to elderly patients. All cases were retrospectively identified based on the co-isolation of *A. baumannii* and *Elizabethkingia* from patients during hospitalization. Among the collected cases, the majority of patients were indeed elderly, with a median age of 70 years (interquartile range, 56-79), which was not a predefined inclusion criterion.

Comment 2: Based on the patient's history, do they reside in a rural or urban area? You noted that mosquitoes transmit *Elizabethkingia*.

Response 2:

All patients included in this study were treated at a tertiary general hospital located in an urban area. However, detailed information regarding long-term residence (rural versus urban) was not available in the medical records. Importantly, although *Elizabethkingia* was originally identified in association with mosquitoes, the infections described in this study occurred in a hospital setting, and mosquito-borne transmission was not involved.

Comment 3: I believe the study should place greater emphasis on the mechanisms underlying the interactions between microorganisms, as mixed infections are known to be challenging to treat.
Response 3:
We fully agree that elucidating the mechanisms underlying interactions among different microbial species is critical for understanding the challenges associated with treating mixed infections. In the revised manuscript, we have further strengthened and clarified the mechanistic interpretation of our findings by integrating existing phenotypic results with genomic and transcriptomic data. Specifically, we expanded the analysis and discussion of pathways related to efflux pump systems, quorum sensing, and stress response, which may collectively contribute to interspecies interactions and enhanced antibiotic tolerance under co-culture conditions. These additions provide a theoretical basis for understanding how microbial interactions can exacerbate the treatment challenges of polymicrobial infections.

Comment 4: In the text under Table 2, line 10, a repetition was noted (from).
Response 4:
The duplicated word has been removed in the revised manuscript.

Comment 5: For bacterial fitness, did you compare the growth of your strains (wild-type and mutated)?
Response 5:
After carefully reviewing the experimental design related to bacterial fitness in our manuscript, we confirm that this study did not involve the construction or use of mutant strains. All experiments were performed using clinical wild-type isolates. Therefore, bacterial fitness was evaluated by comparing growth characteristics under different experimental conditions, including the presence or absence of antibiotics as well as mono- and co-culture settings. These growth curve analyses were used to assess relative fitness under varying conditions rather than the effects of specific genes.

Comment 6: In Figure 5A, a control medium was not used; similarly, for Figures B and C, there was no wild-type bacteria or control medium included to compare with the mutated strains.
Response 6:
For Figure 5A (Figure 5 in the revised manuscript), the primary objective was to compare biofilm formation between monoculture and co-culture at different ratios. Accordingly, the monoculture and co-culture conditions served as mutual controls under identical media conditions.
For Figures 5B and 5C (Figures 7A and 7B in the revised manuscript), as noted above, this study did not involve the construction or use of mutant strains. All experiments were performed using clinical wild-type isolates, and comparisons were therefore made between different culture conditions (monoculture vs. co-culture) of the same wild-type strains, rather than between wild-type and mutant bacteria.

Comment 7: Regarding Figure 6, I suggest that using a 96-well plate is more precise and reliable than a tube biofilm assay. Additionally, you should repeat the experiment comparing the mutated strain with the wild type and include media as a control.
Response 7:
We thank the reviewer for this constructive suggestion. Quantitative biofilm formation was already assessed using a 96-well plate–based crystal violet staining assay, as described in the section "Co-cultivation Enhances Biofilm Formation of AB2-1 and EA2-2" (Figure 5 in the revised manuscript). For Figure 6, tube-based biofilm assays were intentionally used to enable macroscopic visualization of biofilm formation, as the larger surface area facilitates clearer observation of biofilm biomass. As mentioned previously, this study did not involve the construction or use of mutant strains, and all experiments were conducted using clinical wild-type isolates.

Comment 8: In Figure 7, there is no comparison between your strain and the mutated strain, nor is there a control included. Additionally, please consider incorporating images to illustrate the larvae, both live and dead, along with their symptoms observed during the experiments.
Response 8:
As described in the previous response, this study was conducted using clinical wild-type isolates and did not involve the construction or use of mutant strains. In Figure 7 (Figure 8 in the revised manuscript), larvae injected with PBS were included as a negative control group, while larvae infected with AB, EA, and the AB+EA co-infection served as comparative groups under identical experimental conditions. We regret that representative images of live and dead larvae were not retained during the experiments. In this study, larvae were selected to be creamy white in appearance, highly motile, and capable of a strong righting response, corresponding to final-instar larvae approximately 2-3 cm in length and ~300 mg in weight. Larval death was defined by body melanization and the absence of any response to stimulation with blunt forceps. These experimental criteria have been described in the Materials and Methods section of the revised manuscript (lines 662-665 and 670-672).

Comment 9: The following link provides a more accurate method for measuring bacterial growth: https://www.labtools.us/bacterial-cell-number-od600
Response 9:
We thank the reviewer for the helpful reference. We acknowledge that direct cell number determination can provide a more precise estimation of bacterial growth under certain conditions. In this study, bacterial growth was monitored using optical density measurements, which were applied consistently across all experimental groups. Because our analyses focused on relative differences in growth dynamics between monoculture and co-culture conditions rather than absolute cell numbers, this approach was considered appropriate for the objectives of the study.

We would like to again express our sincere appreciation to the reviewers for their thorough and professional assessment of our work. We believe that the revisions have improved the clarity and rigor of the manuscript. While we have made every effort to address all concerns, there may still be aspects that warrant further improvement. We sincerely welcome any additional comments and would be grateful for your continued guidance.

Re: Spectrum03349-25R1 (**Biofilm-mediated Antibiotic Cross-protection: *Acinetobacter baumannii*-driven Enhancement of *Elizabethkingia anophelis***)

Dear Dr. Na Cheng:

Your manuscript has been accepted, and I am forwarding it to the ASM production staff for publication. Your paper will first be checked to make sure all elements meet the technical requirements. ASM staff will contact you if anything needs to be revised before copyediting and production can begin. Otherwise, you will be notified when your proofs are ready to be viewed.

Data Availability: ASM policy requires that data be available to the public upon online posting of the article, so please verify all links to sequence records, if present, and make sure that each number retrieves the full record of the data. If a new accession number is not linked or a link is broken, provide production staff with the correct URL for the record. If the accession numbers for new data are not publicly accessible before the expected online posting of the article, publication may be delayed; please contact ASM production staff immediately with the expected release date.

Publication Fees: For information on publication fees and which article types have charges, please visit our website. We have partnered with Copyright Clearance Center (CCC) to collect author charges. If fees apply to your paper, you will receive a message from no-reply@copyright.com with further instructions. For questions related to paying charges through RightsLink, please contact CCC at ASM_Support@copyright.com or toll free at +1-877-622-5543. CCC makes every attempt to respond to all emails within 24 hours.

ASM Membership: Corresponding authors may join or renew ASM membership to obtain discounts on publication fees. Need to upgrade your membership level? Please contact Customer Service at Service@asmusa.org.

Embargo Policy: A press release may be issued as soon as the manuscript is posted on the Spectrum Latest Articles webpage. The corresponding author will receive an email with the subject line "ASM Journals Author Services Notification" when the article is available online.

The ASM Journals program strives for constant improvement in our submission and publication process. Please tell us how we can improve your experience by taking this quick Author Survey.

Thank you for submitting your paper to Spectrum.

Sincerely,
Valerie Carabetta
Editor
Microbiology Spectrum
